# Identification and validation of LDHA and SLC16A1 for predicting prognosis and diagnosis in lower-grade glioma

**DOI:** 10.1007/s12672-025-03297-2

**Published:** 2025-08-09

**Authors:** Jingyi Huang, Qishun Wang, Zhijun Li, Xuning Huang, Wen Wang, Jiao Liu

**Affiliations:** 1 Human Anatomy and Histoembryology, Shandong Second Medical University, No. 7166 Baotong West Street, Weifang City, 261053 Shandong Province China; 2 Changyi City Center for Disease Control and Prevention, Changyi City, Shandong Province China; 3 Department of Surgery, Changyi City Hospital of Traditional Chinese Medicine, Changyi City, Shandong Province China; 4 Changyi City Hospital of Traditional Chinese Medicine Pharmacy, Changyi City, Shandong Province China; 5 Department of Internal Medicine, Changyi City Hospital of Traditional Chinese Medicine, Changyi City, Shandong Province China; 6 School of Basic Medicine Sciences, Shandong Second Medical University, Weifang City, China

**Keywords:** Lower-grade glioma, *LDHA*, *SLC16A1*, Prognosis, Diagnosis

## Abstract

**Purpose:**

This study aimed to analyze the prognostic and diagnostic value of *Lactate dehydrogenase A (LDHA)* and solute carrier family 16 member 1 (*SLC16A1*) in low-grade gliomas (LGG).

**Methods:**

Gene expression datasets for LGG were downloaded from The Cancer Genome Atlas (TCGA), Chinese Glioma Genome Atlas (CGGA), and Gene Expression Omnibus (GEO) databases. The prognostic value of *LDHA* and *SLC16A1* in LGG was analyzed using the survival package. Receiver operating characteristic (ROC) curves were drawn to evaluate the ability of the model to distinguish between patients with LGG and controls. Gene set enrichment analysis (GSEA) of single gene was utilized to explore the potential biological function of the two genes. The protein levels of *LDHA* and *SLC16A1* were analyzed using the Human Protein Atlas database. *LDHA* and *SLC16A1* expression was verified using real-time reverse transcription polymerase chain reaction. Finally, the effects of low *SLC16A1* expression on the proliferation, migration, and invasion of LGG cells were investigated using CCK-8 and Transwell assays.

**Results:**

*LDHA* was downregulated, and *SLC16A1* was upregulated in LGG tissues compared to normal tissues in TCGA dataset. Kaplan–Meier (K–M) survival and ROC curves revealed that these two genes have potential prognostic and diagnostic performances. *LDHA* positively correlated with *SLC16A1* in TCGA and CGGA cohorts. GSEA demonstrated that *LDHA* is involved in the chemokine and NOD-like receptor signaling pathways, whereas *SLC16A1* is involved in the JAK-STAT and NOD-like receptor signaling pathways. Immunohistochemical profiles of *LDHA* and *SLC16A1* were consistent with their mRNA expression levels. SLC16A1 overexpression and downregulation of *LDHA* have been validated in glioma cell lines. Additionally, low *SLC16A1* expression inhibited the proliferation, migration, and invasion of glioma cells.

**Conclusion:**

*LDHA* and *SLC16A1* have potential prognostic and diagnostic values for LGG. Therefore, *SLC16A1* may serve as a potential biomarker for the diagnosis and treatment of LGG.

**Supplementary Information:**

The online version contains supplementary material available at 10.1007/s12672-025-03297-2.

## Introduction

Gliomas are the most common malignant neural epithelial tumors with high morbidity and recurrence rates. According to the tumor histopathology, gliomas are divided into four World Health Organization (WHO) grades, ranging from benign grade I tumors to highly malignant grade IV gliomas [1]. Low-grade glioma (LGG), including grades I and II as classified by the WHO, is usually slow-growing, intermittently progressive, and invasive, accounting for approximately 22% of all adult brain tumors [2]. However, recent developments have shown that classification is unnecessary and have led to the term “LGG” to designate both grades II and III gliomas [2, 3]. Although epileptic seizures is the most common manifestation in grade II gliomas, some patients with LGG is asymptomatic, which affects patient prognosis [4]. Thus, it is necessary to identify specific biomarkers for the individualized treatment and prognosis of LGG.

In recent years, molecular markers for the diagnosis, prediction, and prognosis of gliomas have been extensively explored. Ali et al. found that miR-29a, miR-106a, and miR-200a can serve as biomarkers for monitoring therapeutic efficacy of patients with glioblastoma (GBM) [5]. Another study confirmed that patients with GBM with IDH1 mutations and MGMT methylation have better survival patterns, and when the two were used in combination, the treatment effect and survival rate improved [6]. Swellam et al. found that NDRG2 methylation in patients with GBM significantly increased with the deterioration of overall survival and progression-free survival, and that NDRG2 methylation levels can serve as an effective biomarker for diagnosis and prognosis [7]. Metabolic reprogramming is a hallmark of cancer [8, 9]. Lactate dehydrogenase A (*LDHA*) is a key glycolytic enzyme that converts pyruvate to lactate in the final step of glycolysis [9, 10]. Lactate has been suggested to promote the survival, invasiveness, resistance, and activation of oncogenic signaling pathways in cancer cells [11]. Lactate is a hydrophilic and weak acid; thus, its transport across membranes requires transporters belonging to the monocarboxylate transporter (MCT) family [12]. It has been reported that lactate uptake is mediated by MCT1 (encoded by *SLC16A1*). MCT1 upregulates the lactic acid exchange [13]. A recent study demonstrated that the growth and proliferation of intestinal and intraductal papillary mucinous neoplasms and hepatocellular carcinoma subtype tumors mainly rely on lactate dehydrogenases (such as *LDHA*) and *SLC16A1* gene expression [14]. However, the roles of *LDHA* and *SLC16A1* in LGG development have not been reported to our best knowledge.

In the present study, we investigated the roles of *LDHA* and *SLC16A1* in LGG. The expression of these two genes in tumor and normal tissues was investigated. Furthermore, the prognostic value of these two genes in LGG was evaluated. We performed gene set enrichment analysis (GSEA) of *LDHA* and *SLC16A1* to investigate the potential mechanisms of the two genes in LGG progression. The protein levels of *LDHA* and *SLC16A1* were analyzed using the Human Protein Atlas (HPA) database, and the differential expression and function of *LDHA* and *SLC16A1* were validated in glioma cell lines.

## Methods

### Data sources

Gene expression data for LGG were obtained from the Cancer Genome Atlas (TCGA) database [15]. Moreover, 105 normal cerebral cortex samples from the Genotype-Tissue Expression (GTEx) database were used as controls. According to the clinical information of the LGG samples downloaded simultaneously, disease samples with prognostic information were retained, and 628 samples were obtained, including 523 LGG samples and 105 controls. Among the 523 LGG samples, 518 had complete survival information and were used for prognostic analyses. Grade I gliomas are rare, and grade IV gliomas present with superior malignancy. The TCGA database classifies grades II and III gliomas as LGG [16]. Thus, LGG was defined as a grade II or III glioma in the following investigation. The inclusion and exclusion criteria are as follows: (1) Samples from patients with grades II and III in TCGA were included, with grades I and IV excluded from the analysis; (2) Standardized patient follow-up data, patients with complete survival information were used for follow-up prognostic analysis; (3) Data sets that include the largest possible sample size; (4) All expression values are log2 logization. The RNA-seq dataset, including 172 LGG samples, was downloaded from the Chinese Glioma Genome Atlas (CGGA) database [17], and the survival information of the patients was downloaded to analyze the prognosis of *LDHA* and *SLC16A1*. Two expression datasets, GSE15824 and GSE16011, were downloaded from the Gene Expression Omnibus (GEO) database. GSE15824 included 45 samples (five control samples and 40 tumor samples from 26 GBM and 14 LGG), which were sequenced on a GPL570 ([HG-U133_Plus_2] Affymetrix Human Genome U133 Plus 2.0 Array. GSE16011contained 284 cases (eight control samples, 117 LGG, and 159 GBM samples), and the sequencing platform used was GPL8542 (Affymetrix GeneChip Human Genome U133 Plus 2.0 array [CDF: Hs133P_Hs_ENTREZG.cdf]). Owing to the lack of survival information, the GSE15824 and GSE16011 datasets were only used for expression pattern and diagnostic value analysis of *LDHA* and *SLC16A1*. The clinicopathological features of the patients were shown in Table 1. Details of the sample size are shown in Table 2.

Download the mRNA probe expression matrix file corresponding to each dataset, and download the annotation file corresponding to the sequencing platform. Converts probes to gene symbol one by one, removing probes that do not match the gene symbol. For different probes mapped to the same gene, the average value was taken as the expression value of the gene, that is, the gene expression matrix was obtained for subsequent analysis.

**Table 1:** The clinicopathological features of the patients

Cohort	TCGA-LGG(*n* = 518)	CGGA-LGG(*n* = 172)
Gender		
Male	287	106
Female	231	66
Age(years)		
< 60	448	160
≥ 60	70	12
Race		–
White	477	
Black or African American	22	
Asian	8	
American Indian or Alaska native	1	
Not reported	10	
Vital_status		
Alive	388	82
Dead	130	90
Primary_diagnosis.diagnoses		–
Mixed glioma	130	
Oligodendroglioma, NOS	114	
Astrocytoma, NOS	66	
Astrocytoma, anaplastic	129	
Oligodendroglioma, anaplastic	79	
IDH		
Mutant	407	127
Wildtype	111	44
NA	–	1

**Table 2:** Details of sample size

Database	Samples
TCGA	523 LGG samples (518 samples had survival information for prognostic analysis)
GTEx	105 control samples
CGGA (CGGA-325)	172 LGG samples (Prognostic analysis)
GEO (GSE15824)	5 control samples, 26 GBM samples, 14 LGG samples (Gene expression and diagnostic analysis)
GEO (GSE16011)	8 control samples, 159 GBM samples, 117 LGG samples (Gene expression and diagnostic analysis)

### Expression profiles of *LDHA/SLC16A1* in LGG and controls

The expression levels of *LDHA* and *SLC16A1* in the TCGA + GTEx, GSE15824, and GSE16011 cohorts were analyzed. Differences between *LDHA* and *SLC16A1* in the tumor vs. normal groups were analyzed using the Wilcoxon test in the R language.

### Prognostic value of target genes in LGG

Based on the TCGA-LGG and CGGA-325 datasets, the tumor samples were classified into high- and low-expression groups according to the median values of *LDHA* and *SLC16A1* gene expression [18, 19]. Kaplan–Meier (K–M) survival analysis was conducted using survival 2.41-1 [20] to analyze the relationship between gene expression and LGG patient survival. The combined effects of *LDHA* and *SLC16A1* on LGG cell survival of LGGs was also investigated.

### Receiver operating characteristic (ROC) curve of *LDHA* and *SLC16A1*

To analyze the diagnostic value of *LDHA* and *SLC16A1* in LGG, ROC curves were plotted for the TCGA-LGG, GSE15824-LGG, and GSE16011-LGG cohorts using the R package pROC 1.18.0 [21]. The area under the ROC curve (AUC) was calculated to evaluate the ability of the model to distinguish between patients with LGG and controls. The AUC is between 0 and 1, and the greater the AUC value (the closer it is to 1), the better are the diagnostic results.

### Gene set enrichment analysis (GSEA) of *LDHA* and *SLC16A1*

To explore the correlation between *LDHA* and *SLC16A1* in LGG, Spearman’s correlation analysis was employed based on TCGA and CGGA datasets using R software. With MSigDB v7.1 [22] database c2.cp.kegg.v7.4. symbols.gmt as the enrichment background, the expression profile of TCGA-LGG patients was analyzed using GSEA. The significant Kyoto Encyclopedia of Genes and Genomes pathways enriched by *LDHA* and *SLC16A1* were analyzed, and pathways with *P* < 0.05 were presented. 

### Protein expression of *LDHA* and *SLC16A1* based on the HPA database

The HPA database is a collection of protein expression in 48 human normal tissues, 20 tumor tissues, and 64 cell lines through immunoassays [23]. Immunochemistry images of *LDHA* and *SLC16A1* in the normal cerebral cortex and glioma tissues were retrieved from the HPA online database.

### Cell culture and processing

Normal human glial cells were purchased from Shanghai Cell Bank of the Chinese Academy of Sciences (Shanghai, China). The LGG cell lines of SW1088 and HS683 cell lines were obtained from the American Type Culture Collection (ATCC, USA). Cells were cultured in Dulbecco’s modified Eagle’s medium (Gibco, USA) supplemented with 10% fetal bovine serum (Gibco, Grand Island, NY, USA) and maintained at 37 °C under 5% CO_2_. *SLC16A1* small interference RNA (si - SLC16A1) and the negative control (si - NC) sequence (Gemma, Shanghai) was as follows: si-SLC16A1:5’- GAGGAAGAGACCAGTATAGATGTTGCTGG-3; si-NC: 5’- CCGCCCTTTTTTGGGCCTAAAACCCCTGAATAGTCCG − 3. ’ si-SLC16A1 and si-NC were transfected into LGG cells lines by Lipofectamin3000 and continued cultured for 48 h.

### Real time PCR assay

Real-time reverse transcriptase-polymerase chain reaction (RT-qPCR) was used to validate the relative mRNA levels of *LDHA* and *SLC16A1.* Total RNA was extracted using the TRIzol reagent (Invitrogen, USA). cDNA was generated using MMLV reverse transcriptase (Promega, USA). RT-qPCR was performed using a LightCycler 480II qRT-PCR instrument (Roche, Switzerland). The sequences of primer pairs: *LDHA*: F: 5′-CTCCTGTGCAAAATGG-CAAC-3′, R: 5′-CCTAGAGCTCACTAGT-CACAG-3′; *SLC16A1*: F: 5′-CCATTGTGGAATGCGTCCT-3’, R: 5′-CCTACTTCTTTCCCCCATCC-3′. Actin was used as an internal reference. The relative expression of each gene was quantitatively analyzed using the ΔΔC_t_ method.

### Cell proliferation analysis

Glioma cells in the logarithmic growth phase were selected and seeded in 96-well plates at a density of 4 × 10^3^ cells/well. Three wells were used for each group. After cultivation, 10-µL CCK-8 solution was added to each well and continue incubating for 4 h, and the absorbance (A) value at 450 nm was detected on the microplate reader (Thermo Fisher, USA).

### Transwell migration and invasion assays

Uniform Matrigel (BD, USA) was added to the upper layer of the basement membrane of the Transwell chamber (Millipore, Billerica, MA, USA), and 100-µL cell suspension was spread to the upper chamber of the Transwell (5 × 10^4^ cells/chamber). A total of 600 µL of medium containing 10% FBS was aspirated and added to the lower Transwell chamber. After 24 h of cell culture, the chambers were removed and fixed in 4% paraformaldehyde for 30 min, followed by crystal violet staining (Beyotime, Hangzhou, China) for 30 min. Migration results were observed under a microscope. For the invasion experiment, the BD Matrigel™ Matrix was frozen thawed and diluted with serum-free medium at a ratio of 1:2 and then coated onto the bottom membrane of the chamber and incubated at room temperature for 1 h. After rinsing with serum-free medium, 70 µL of serum-free medium was added and incubated at 37℃ for 30 min, and the remaining experimental procedures were similar to the migration assay.

### Western blot

48 h after transfection, cells were collected and lysed with RIPA cell lysate to extract total cell protein. The protein concentration was determined by BCA method, then 30 µg protein per well was deposited on PVDF membrane by electrophoresis, and enclosed in 5% skim milk at room temperature for 1 h. Mouse anti-human SLC16A1 monoclonal antibody diluted at 1:500 and mouse anti-human GAPDH monoclonal antibody diluted at 1:1000 were added (as internal reference), respectively, and the reaction was overnight at 4 ℃. After TBST rinsing, secondary antibody (1:2000) was added and reacted at 37 ℃ for 1.5 h. And finally visualized using an enhanced chemiluminescence system (BioRad, Hercules, CA, USA).

### Statistical analysis

Data analysis was conducted using GraphPad Prism Software and R packages. The list of R packages implemented in this study is shown in Table 3. Data are presented as mean ± standard deviation, and statistical analysis was conducted using a two-tailed Student’s t-test. Differences were considered statistically significant at *P* < 0.05.

**Table 3:** R packages and versions implemented in this study

*R* package	Version
Survival	2.41-1
pROC	1.18.0
ggplot2	3.5.1
clusterProfiler	4.12.6

## Results

### Expression difference of transporters (*LDHA*/*SLC16A1*) in LGG and normal tissue

**Fig. 1:**
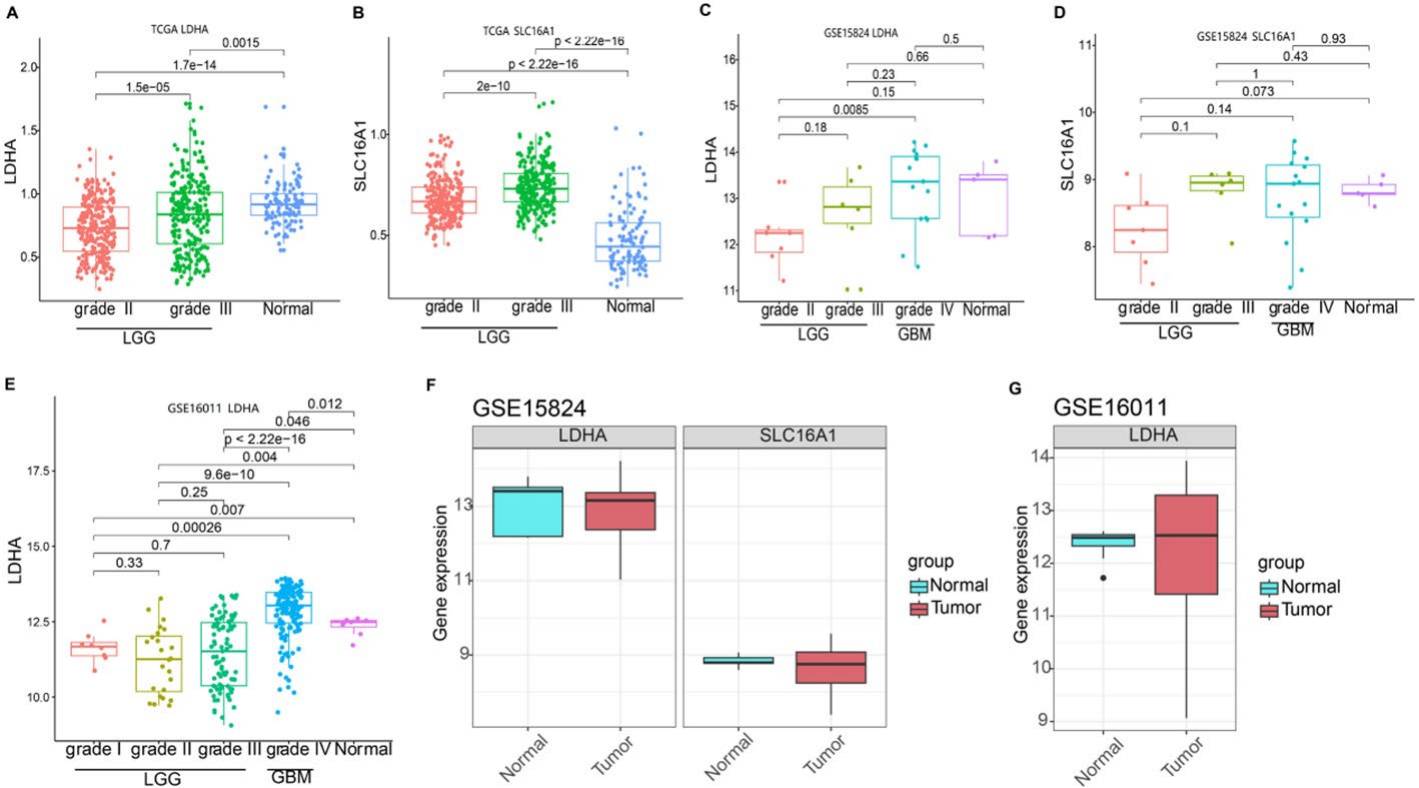
Differential expression of *LDHA/SLC16A1* in LGG and normal tissue. Box plot of *LDHA* (**A**) and *SLC16A1* expression (**B**) between tumor and normal groups in TCGA-LGG dataset. Box plot of *LDHA* (**C**) and *SLC16A1* expression (**D**) according to the grading based on GSE15824 dataset. Box plot of *LDHA* expression (**E**) according to the grading based on GSE16011. (**F**) The box plot of *LDHA* and *SLC16A1* expression in GSE15824 LGG cohort. (**G**) Box plot of *LDHA* expression of GSE16011 LGG cohort. * indicated *P* < 0.05

The expression levels of *LDHA* and *SLC16A1* in LGG and control samples were extracted from TCGA and GTEx expression profile datasets, and the expression differences of *LDHA* and *SLC16A1* in the tumor vs. normal groups were analyzed by R Wilcoxon test according to grading. Based on the TCGA dataset, *LDHA* was significantly downregulated and *SLC16A1* was significantly up-regulated in the LGG (grade II and grade III) groups compared to the normal controls (*P* < 0.001, Fig. 1A and B). The expression levels of *LDHA* and *SLC16A1* in GSE15824 and GSE16011 of all tumor samples (grades I–IV) were also extracted. The expression of *LDHA* and *SLC16A1* in all grade tumor samples (grades I to IV) in the GSE15824 dataset was not significantly different from that in the control group (*P* > 0.05, Fig. 1C and D), and the expression of *LDHA* in grade I to grade IV in the GSE16011 dataset was significantly lower than that in the control group (all *P* < 0.05, Fig. 1E). *SLC16A1* was not annotated based on the GPL8542 platform, and its expression profile of *SLC16A1* was obtained from the GSE16011 dataset. Furthermore, we analyzed the expression profiles of *LDHA* and *SLC16A1* in LGG samples from the GSE15824 (14 LGG and 5 control) and GSE16011 (117 LGG and 8 control) datasets. *LDHA* expression in LGG was relatively low in the GSE15824 (*P* > 0.05, Fig. 1F) and GSE16011 datasets (*P* < 0.05, Fig. 1G), which was consistent with the results of the *LDHA* expression trend based on the TCGA dataset. However, there was no significant change in the expression of *SLC16A1* in the GSE15824 dataset (*P* > 0.05, Fig. 1F). We observed diverse *LDHA* expression in LGG, possibly due to the heterogeneity of the tumor samples and differences in tumor purity‌.

### Prognostic value of *LDHA*/*SLC16A1* in glioma

**Fig. 2:**
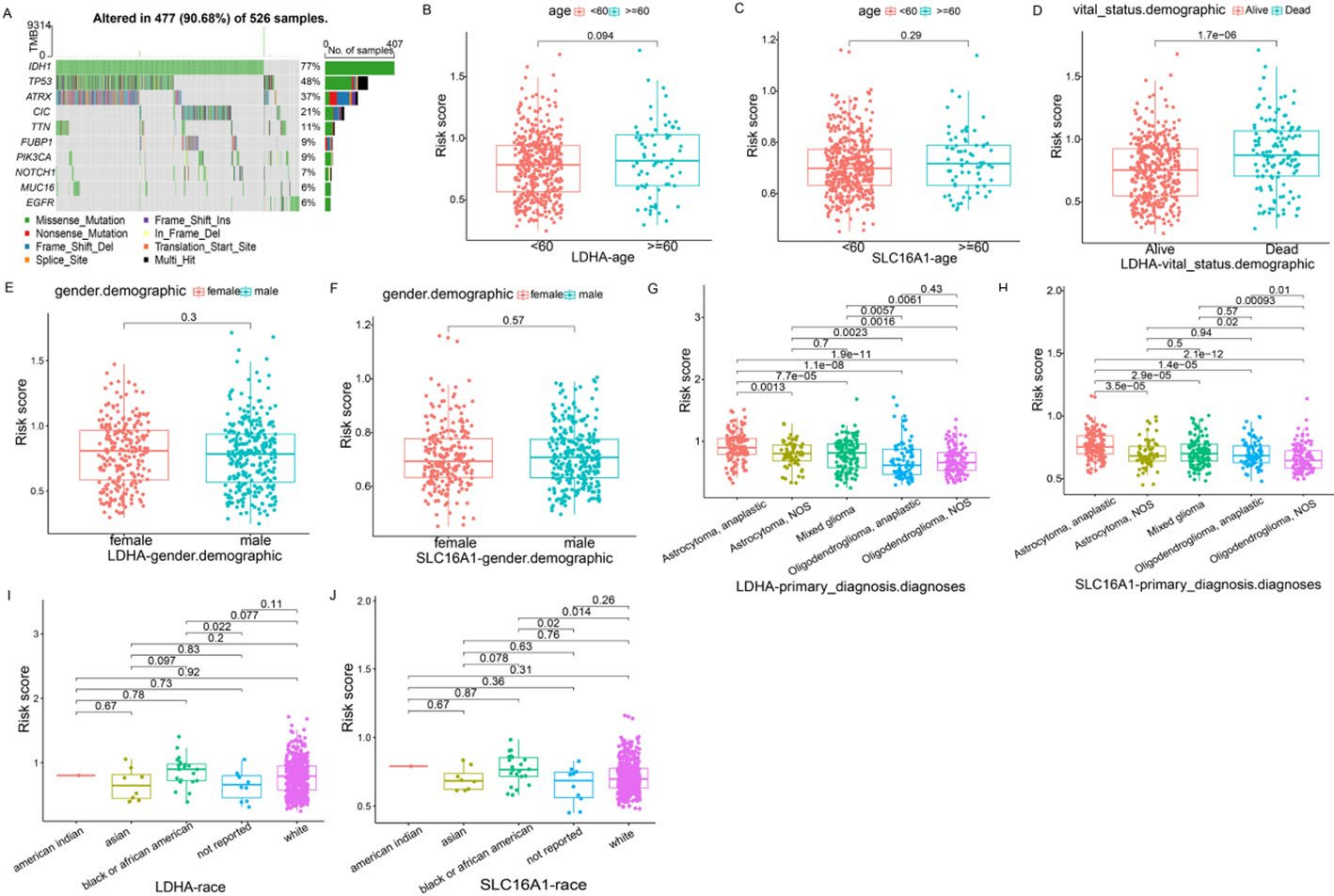
Correlation between gene expression and clinical features. Waterfall diagram of patient IDH status (**A**). Correlation of LDHA expression with age (**B**), vital status (**D**), gender (**E**), primary diagnosis (**G**), and race demographic (**I**). Correlation between the expression of SLC16A1 and age (**C**), gender (**F**), primary diagnosis (**H**), and race demographic (**J**)

In this study, we also analyzed the IDH status of the patients, as shown in Fig. 2A. It can be seen that the mutation frequency of IDH accounts for 77%. In addition, we also analyzed the correlation between the expression of LDHA and SLC16A1 and clinical features such as age, gender, primary diagnosis, and race demographic. The results showed that the expression of LDHA and SLC16A1 was significantly correlated with primary diagnosis, but not with other clinical features (Fig. 2B-J).

**Fig. 3:**
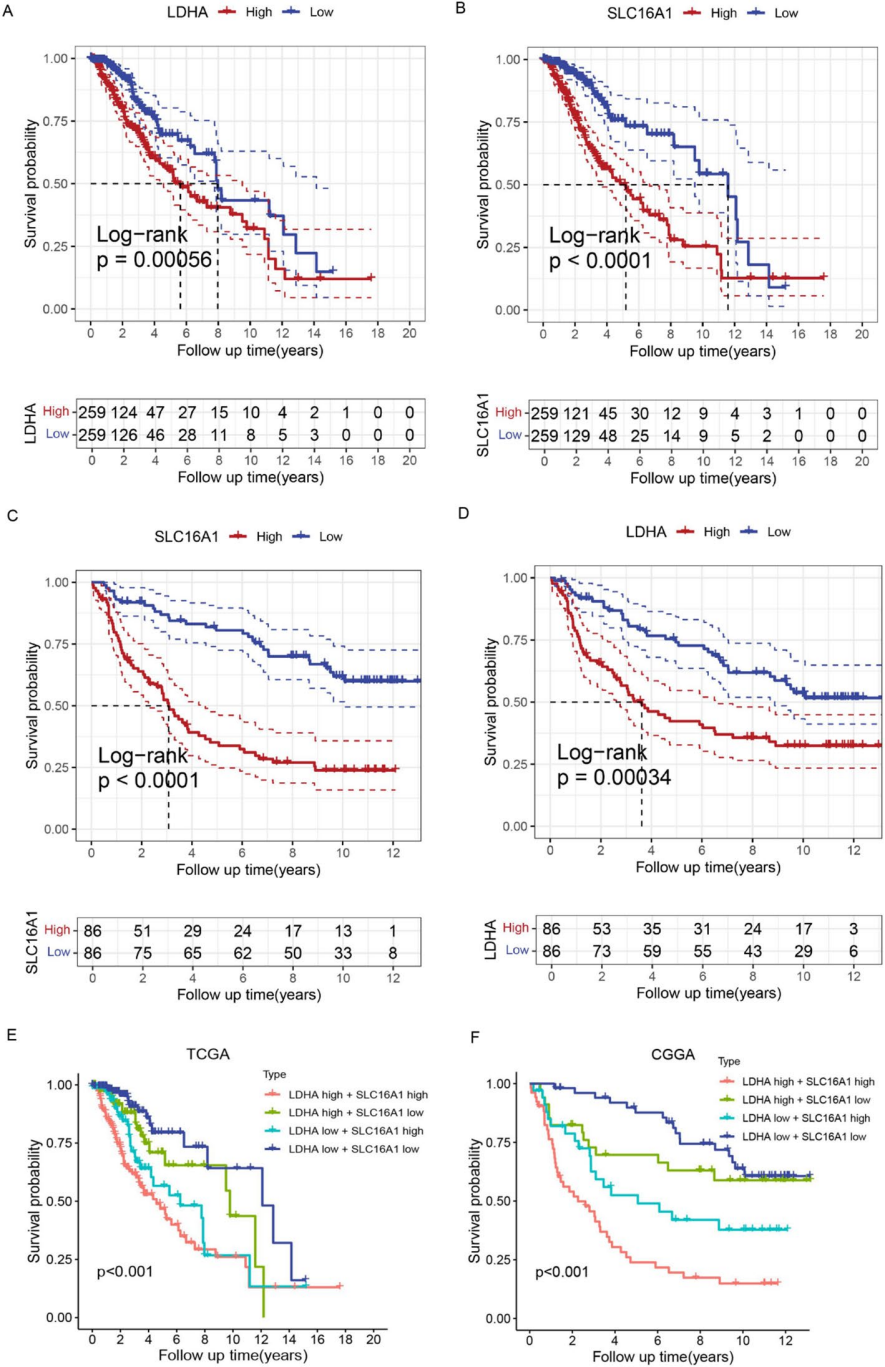
Prognostic value of *LDHA*/*SLC16A1* in LGG. K-M survival curves of *LDHA* (**A**) and *SLC16A1* (**B**) in TCGA-LGG dataset. K-M survival curves of *LDHA* (**C**) and *SLC16A1* (**D**) in CGGA LGG samples. The combined effect of *LDHA* and *SLC16A1* expression on survival rate in TCGA (**E**) and CGGA cohort (**F**)

The correlation between *LDHA*/*SLC16A1* expression and prognosis was analyzed using survival analysis in TCGA and CGGA datasets. K–M survival curves demonstrated that high expression of *LDHA* and *SLC16A1* was significantly correlated with poor outcomes of LGG patients in TCGA cohort (*n* = 518, *P* < 0.001, Fig. 3A and B). We observed similar results in the CGGA cohort, where high expression of *LDHA* and *SLC16A1* was significantly associated with poor outcomes in patients with LGG (*n* = 172, *P* < 0.001, Fig. 3C and D). Furthermore, the prognostic value of the combination of *LDHA* and *SLC16A1* was analyzed. As depicted in Fig. 3E and F, patients with high expression of *LDHA* and *SLC16A* correlated with worse prognosis, while those with low *LDHA* and *SLC16A* expression displayed the best outcomes compared with the other groups (all *P* < 0.001).

### The diagnostic value of *LDHA*/*SLC16A1* in glioma

To further illustrate the diagnostic value of *LDHA/SLC16A1* in LGG, ROC curves were established. As shown in Supplemental Fig. 1A, in the TCGA-LGG, the AUC values of the two genes were > 0.65, indicating that *LDHA* and *SLC16A1* have potential diagnostic value in LGG. Moreover, LGG samples from the GSE15824 and GSE16011 datasets were collected for ROC analysis. The results showed that the AUC values were 0.686 for *LDHA* and 0.629 for *SLC16A1* in the GSE15824 LGG cohort and 0.751 for *LDHA* in the GSE16011 LGG cohort (Supplemental Fig. 1B and C). This suggests that these two genes have better diagnostic value for LGG.

### Correlation and biological function of *LDHA* and *SLC16A1*

**Fig. 4:**
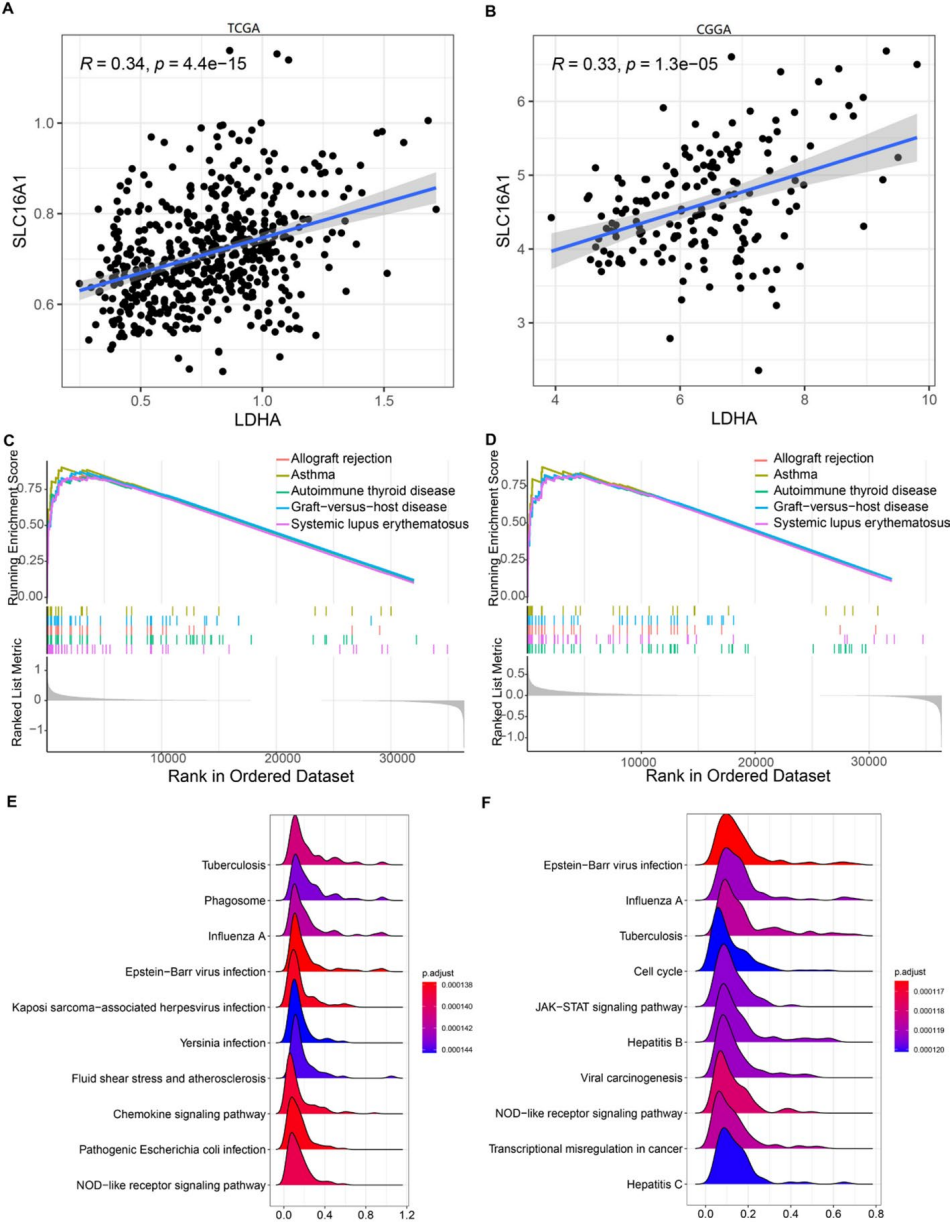
Correlation between *LDHA* and *SLC16A1* and their potential biological functions. Pearson correlation analysis between *LDHA* and *SLC16A1* in TCGA (**A**) and CGGA (**B**). By GSEA analysis, the top5 pathways ranked by enrichmentScore for *LDHA* (**C**) and *SLC16A1* (**D**). The top10 pathways ranked by *P* value for *LDHA* (**E**) and *SLC16A1* (**F**)

In the TCGA cohort, *LDHA* expression was positively correlated with *SLC16A1* expression (*R* = 0.34, *P* < 0.05). Similarly, *LDHA* expression positively correlated with *SLC16A1* expression in the CGGA dataset (*R* = 0.33, *P* < 0.05) (Fig. 4A and B). Furthermore, the GSEA algorithm was used to identify the differential regulatory pathways between the high- and low-expression groups of *LDHA* and *SLC16A1* based on the TCGA dataset. The pathways closely related to the high-expression groups were considered activated pathways. The top five pathways ranked by enrichment score are revealed in Fig. 4C and D, and the top10 pathways ranked by *P*-value are presented in Fig. 4E and F. For both genes, allograft rejection, autoimmune thyroid disease, asthma, systemic lupus erythematosus, and graft-versus-host disease were activated in the high-expression groups. In the ridge maps, *LDHA* was closely related to the chemokine and NOD-like receptor signaling pathways. *SLC16A1* is involved in JAK-STAT and NOD-like receptor signaling pathways.

### Protein expression of *LDHA* and *SLC16A1* based on the HPA database

**Fig. 5:**
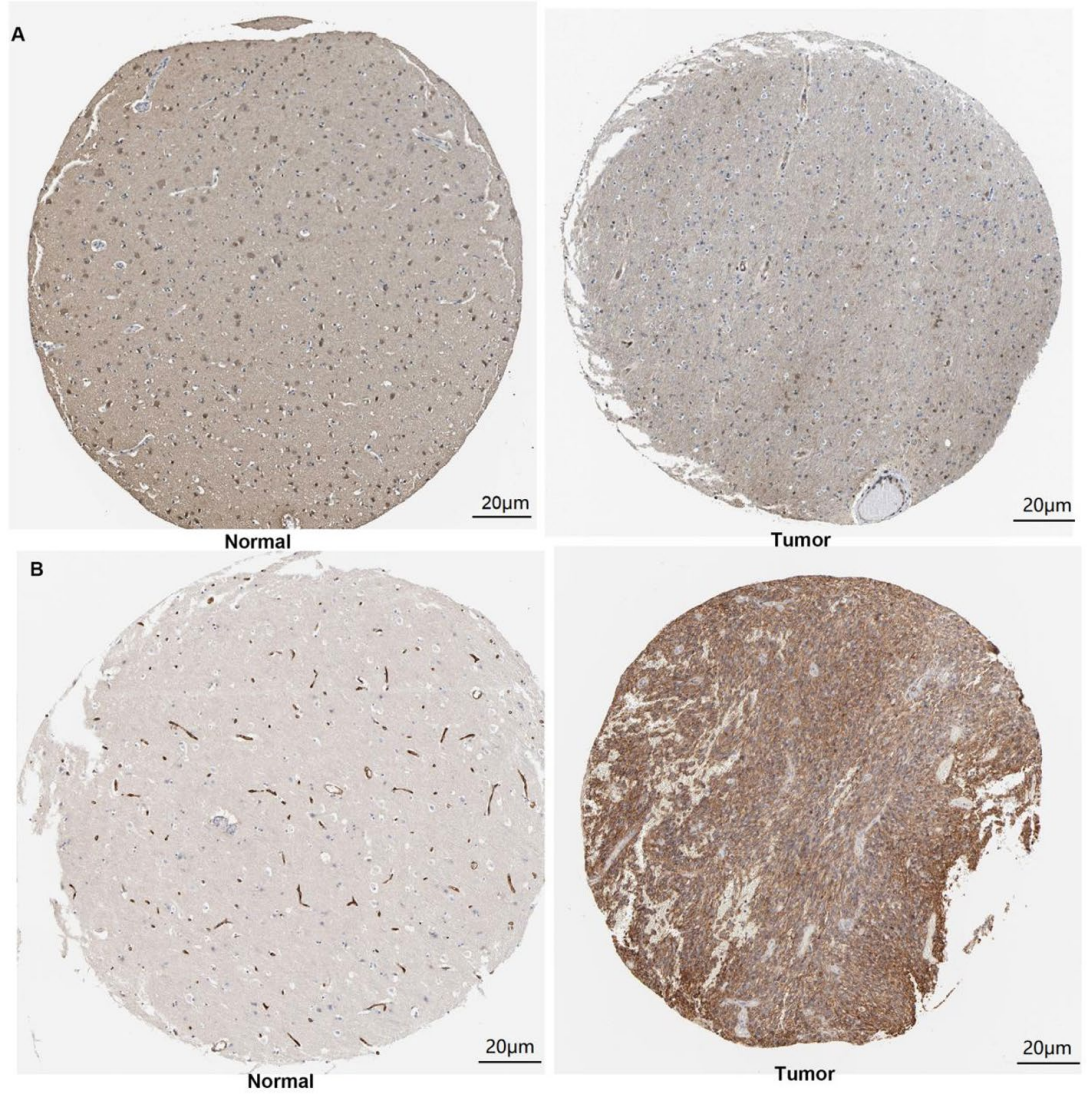
Immunohistochemical images of *LDHA* and *SLC16A1* based on HPA database. Immunohistochemical results of (**A**) *LDHA* and (**B**) *SLC16A1* in normal and tumor tissues. For *LDHA* (normal: CAB015336, Female, age 54, Cerebral cortex (T-X2020), Normal tissue, NOS (M-00100), Patient id: 2523, Intensity: Negative, Quantity: None, Staining: Low, Intensity: Moderate, Quantity:<25%; Tumor: Glioma, CAB015336, Female, age 36, Cerebral cortex (T-X2020), Glioma, malignant, High grade (M-938033), Patient id: 1587, Tumor cells, Staining: Low, Intensity: Moderate, Quantity:<25%, Location: Cytoplasmic/membranous); For *SLC16A1* (normal: Cerebral cortex, HPA003324, Male, age 45, Cerebral cortex (T-X2020), Normal tissue, NOS (M-00100), Patient id: 2521, Endothelial cells, Staining: High, Intensity: Strong, Quantity:>75%, Location: Cytoplasmic/membranous; Tumor: Glioma, HPA003324, Male, age 56, Cerebral cortex (T-X2020), Glioma, malignant, High grade (M-938033), Normal tissue, NOS (M-00100), Patient id: 1578, Tumor cells, Staining: High, Intensity: Strong, Quantity: >75%, Location: Cytoplasmic/Membranous)

The immunohistochemical images of *LDHA* and *SLC16A1* in normal cerebral cortical and glioma tissues were retrieved from the HPA database. As shown in Fig. 5, the protein level of *LDHA* was relatively low and *SLC16A1* was higher in tumor tissues than in controls. The patient’s organizational characteristics are shown in Table 4.

**Table 4:** Organizational characteristics of patients

	LDHA		SLC16A1	
Patient ID	2523	1587	2521	1578
Age (years)	54	36	45	56
Gender	Female	Female	Male	Male
Sample type	Normal sample	Glioma sample	Normal sample	Glioma sample
Location	Cerebral cortex (T-X2020)	Cerebral cortex (T-X2020)	Cerebral cortex (T-X2020)	Cerebral cortex (T-X2020)
Stained area	Cytoplasmic/membranous	Cytoplasmic/membranous	Cytoplasmic/membranous	Cytoplasmic/membranous
Staining	Low	Low	High	High
Intensity	Moderate	Moderate	Strong	Strong
Quantity	< 25%	< 25%	> 75%	> 75%

### Low expression of *SLC16A1* inhibits proliferation, migration, and invasion of glioma cells

**Fig. 6:**
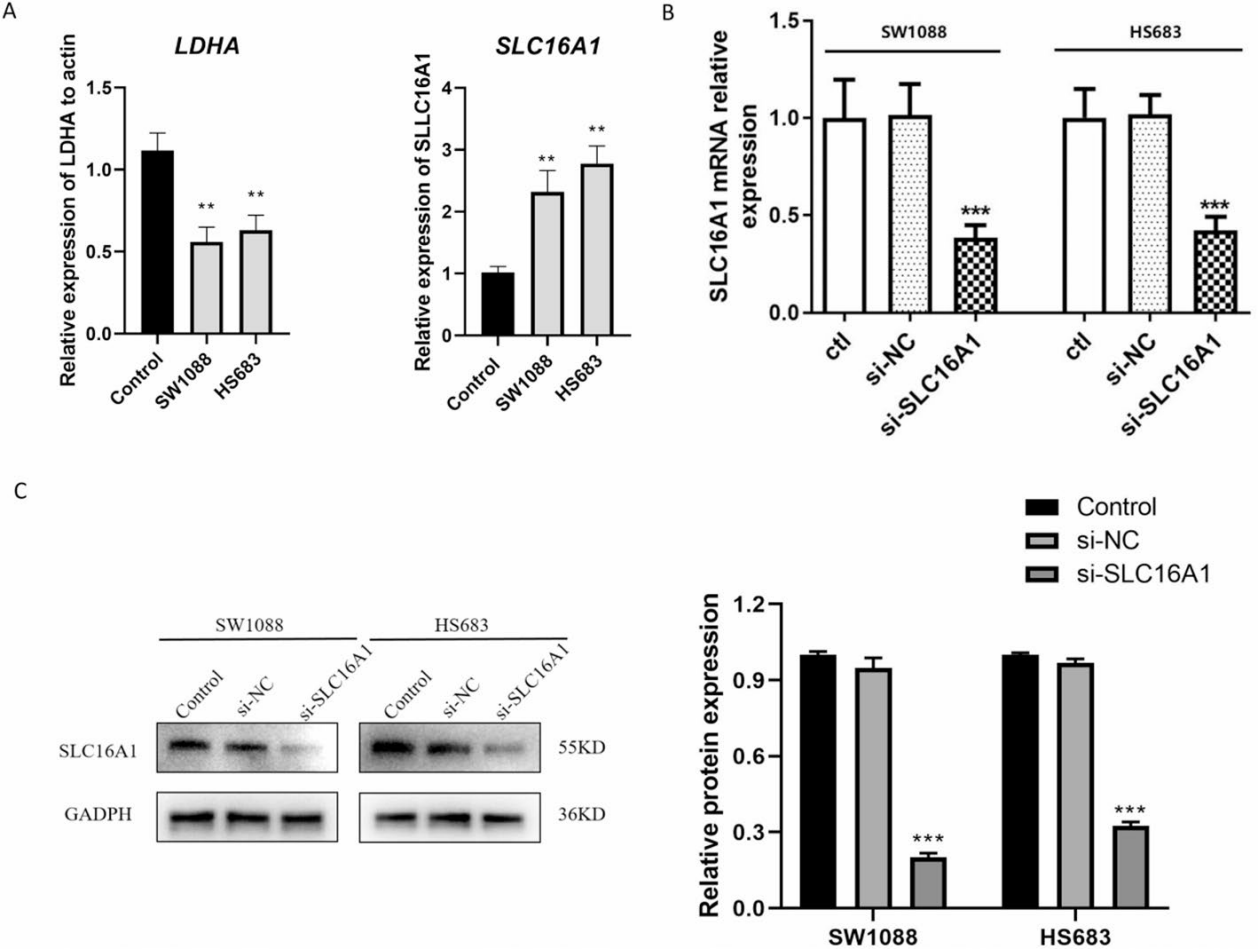
Expression of *SLC16A1* in SW1088 and HS683 after transfection with si-SLC16A1. **A** The expressions of LDHA and SLC16A1 in low-grade glioma cell lines. The X-axis represented different cells, while the Y-axis represented the relative expression of LDHA and SLC16A1. Control, normal human glial cells; Low-grade glioma cell lines SW1088 and HS683. **, *P* < 0.01, when compared with control. **B** The mRNA expression of SLC16A1 was detected by qRT-PCR. **C** The protein expression of SLC16A1 was detected by western blot. Protein concentrations were determined by BCA assay, and equal amounts (30 µg) were loaded in each lane.***, *P* < 0.001

**Fig. 7:**
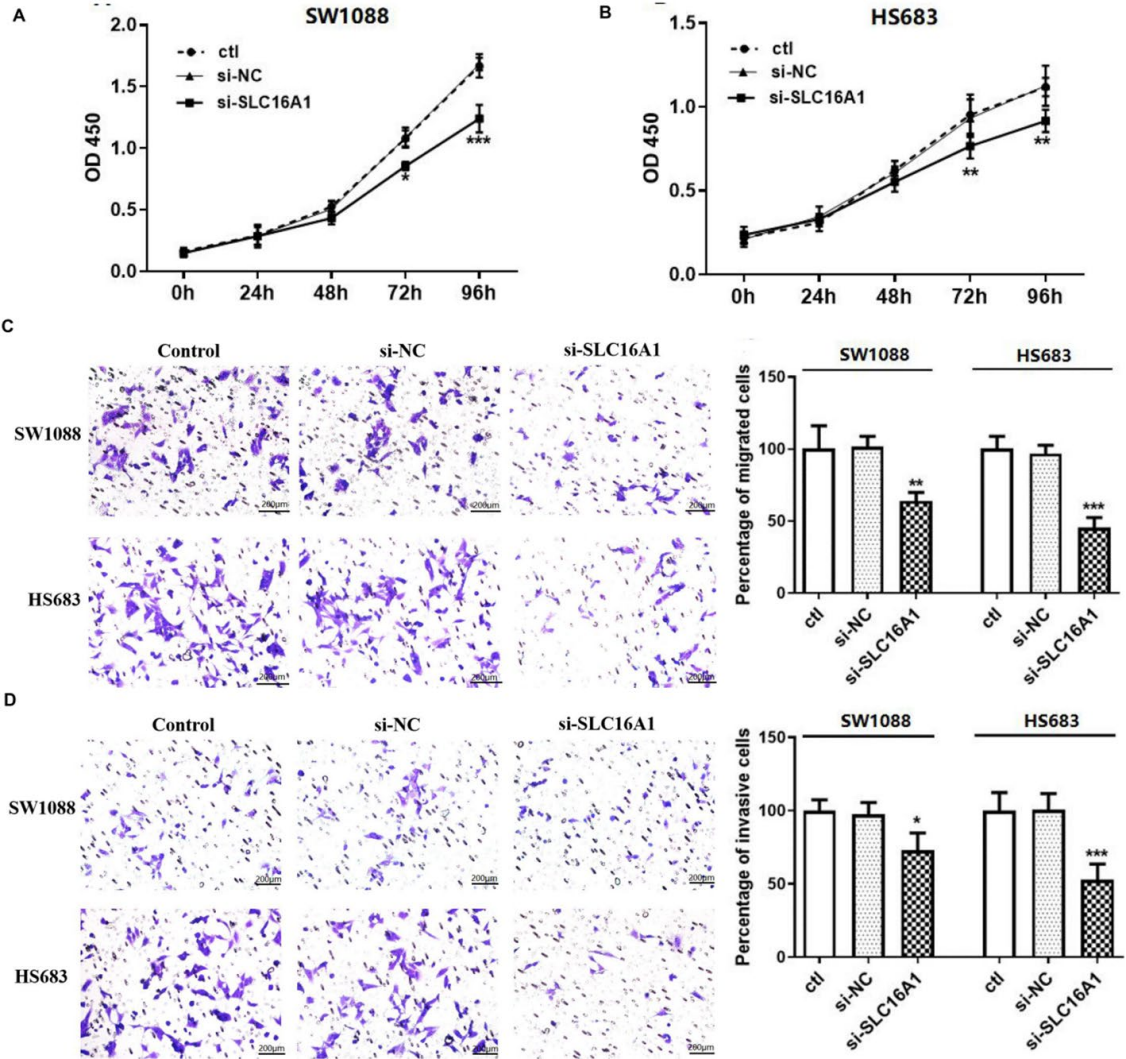
Low expression of *SLC16A1* inhibits glioma cell proliferation, migration, and invasion. **A** Proliferation curve of SW1088 cells. **B** Proliferation curve of HS683 cells. **C** Transwell assay was used to detect the migration ability of SW1088 and HS683 cells (×100). **D** Transwell assay was used to detect the invasion ability of SW1088 and HS683 cells (×100). *, *P* < 0.05, **, *P* < 0.01, ***, *P* < 0.001

The expressions of *LDHA* and *SLC16A1* were validated in LGG cell lines. As illustrated in Fig. 6A, *LDHA* was aberrantly de-expressed and *SLC16A1* was significantly upregulated in LGG cells, which was consistent with the results of the bioinformatics analysis. *SLC16A1* was selected for subsequent experiments. si-SLC16A1 was transfected into SW1088 and HS683 cells, and the mRNA and protein expression levels of *SLC16A1* in SW1088 and HS683 cells were significantly reduced (Fig. 6B and C), indicating successful transfection of si-SLC16A1. Proliferation analysis of CCK-8 demonstrated that the proliferative capacity of SW1088 and HS683 cells was reduced after transfection with si-SLC16A1 (Fig. 7A and B). Moreover, Transwell analysis revealed that low expression of *SLC16A1* inhibited the migration and invasion abilities of SW1088 and HS683 cells compared to the negative control group (Fig. 7C and D).

## Discussion

Lactate secreted by tumors, produced from aerobic glycolysis in cancer cells, is associated with an elevated incidence of metastasis, angiogenesis, and metabolic reprogramming in adjacent tissues. *LDHA* plays a critical role in human cancers owing to its role in promoting glycolysis and converting pyruvate to lactate [24]. Lactate transport across the membrane requires MCT1. Thus, the contribution of *LDHA* and *MCT1* (*SLC16A1*) to tumor progression deserves attention. In this study, by downloading glioma-related datasets from TCGA, CGGA, and GEO, we analyzed the expression, prognosis, and diagnostic values of *LDHA* and *SLC16A1* in LGG cohorts. The results showed that *LDHA* was downregulated and *SLC16A1* was upregulated in LGG tumor tissues compared to normal tissues in TCGA dataset. The protein expression of *LDHA* and *SLC16A1* based on the HPA database was consistent with the mRNA expression levels. QRT-PCR also showed a similar trend. Furthermore, low *SLC16A1* expression inhibited the proliferation, migration, and invasion of SW1088 and HS683 cells. K–M survival curves revealed that high expression of *LDHA* and *SLC16A1* predicted poor prognosis in patients with LGG. ROC curve showed that the two genes exerted potential diagnostic value. GSEA revealed that *LDHA* is involved in the chemokine and NOD-like receptor signaling pathways, whereas *SLC16A1* is involved in the NOD-like receptor and JAK-STAT signaling pathways.

Lactate is the final metabolite of highly proliferative tumors, including GBM, which can be transported to the tumor microenvironment and mediate the survival of tumor cells via MCTs, members of the SLC16 family [25]. MCT1 encoded by *SLC16A1* exerts functions in L-lactate transport to maintain energy balance [26]. In tumor cells, *SLC16A1* is highly expressed during the transport of L-lactate derived from glycolysis. MCTs and L-lactate accumulate and contribute to tumor development and progression [27]. MCT1 is overexpressed in GBM and has been suggested as a potential therapeutic target. In the present study, *SLC16A1* was upregulated in LGG. Although little is known regarding the role of MCT1 in LGG, we infer that *SLC16A1* upregulation plays a key role in the LGG development and progression.

Furthermore, suppressing *LDHA* expression leads to diminished glycolysis, cell growth, and invasion, while increasing apoptosis [28]. As reported in an study in vivo, inhibition of MCT function considerably reduced glioma invasion [25]. Interestingly, in the present study, *LDHA* encoding lactate dehydrogenase A was downregulated in LGG tumor tissues, which may cause less lactate accumulation, leading to reduced growth and invasion of LGG, despite the MCT1 (*SLC16A1*) upregulation. Recently, enzymes associated with catabolism have emerged as prognostic biomarkers of tumors. Zhang et al. found that the cyclin-dependent kinase regulatory subunit 2 is highly expressed in various malignancies and is an independent prognostic factor in HCC [29]. Flap endonuclease 1 is overexpressed in HCC and has been implicated in the progression and metastasis of tumor cells [30]. Overall, *LDHA* and *SLC16A1* are potential targets for LGG therapy.

We also explored the prognostic and diagnostic value of *LDHA* and *SLC16A1* in patients with LGG. The high expression of *LDHA* and *SLC16A1* indicated poor prognosis in LGG, which is in accordance with previous studies [31–34]. For instance, Girgis et al. [31] reported that *LDHA* is a potential prognostic biomarker of clear cell renal cell carcinoma, and the upregulation of *LDHA* indicates poor prognosis. A recent study by Dong et al. [32] also demonstrated that high expression levels of *LDHA* are implicated in the poor differentiation of pancreatic adenocarcinoma, leading to poor survival outcomes. Zhang et al. [33] indicated that high expression of *SLC16A1* predicts poor prognosis in urological cancers. A recent study revealed that the mRNA level of *SLC16A1* was significantly increased in high-grade gliomas compared to LGG and non-tumor controls, suggesting that *SLC16A1* expression is positively correlated with WHO pathological grading and poor survival of gliomas [34]. To the best of our knowledge, this is the first study to demonstrate the diagnostic and prognostic value of *LDHA* and *SLC16A1* in LGG.

To explore the potential mechanisms of these two genes, we conducted GSEA. *LDHA* is involved in chemokine and NOD-like receptor signaling pathways, whereas *SLC16A1* is involved in JAK-STAT and NOD-like receptor signaling pathways. The chemokine system controls almost all types of leukocyte trafficking in the immune system. Chemokines and their receptors are upregulated in numerous human cancers, including gliomas [35, 36]. A pan-cancer analysis by Neapolitan et al. [37] reported that the chemokine signaling pathway is notable in LGG. The JAK-STAT signaling pathway is the response of the cell membrane to extracellular growth factors and cytokines that transmit signals from the cell membrane to the nucleus. Activation of this pathway is effective in predicting the clinical prognosis of glioma [38]. Interestingly, both *LDHA* and *SLC16A1* are involved in the NOD-like receptor signaling pathway. NOD-like receptors are essential for sensing pathogens and risk-associated molecular patterns. NLR signaling dysregulation is critical for the pathogenesis of neurodegenerative and autoimmune diseases and cancers [39]. Notably, NOD-like receptors play a key regulatory role in angiogenesis [40], which is associated with the pathogenesis of gliomas [39]. Glioma growth is closely associated with the brain vascular structure. Once the diameter of the primary tumor reaches 1–2 mm, the cerebrovascular system is destroyed. Thus, tumor growth and invasion in gliomas are marked by angiogenesis [41]. Nevertheless, the exact mechanisms underlying angiogenesis and the expression of these two genes require further investigation. Altogether, we speculate that *LDHA* and *SLC16A1* may be implicated in LGG pathogenesis via the signaling pathways described above.

Overall, this study suggests that *SLC16A1* has a potential prognostic and diagnostic value for LGG. This study also shows that low expression of *SLC16A1* inhibits the biological processes of SW1088 and HS683 cells and can be used as a potential diagnostic and therapeutic target for LGG. The current study used advanced analytical methods and molecular techniques (bioinformatic analysis combined with experimental validation) to identify new targets for the diagnosis and prognosis of LGG, laying the foundation for the development of personalized treatment for patients.

## Conclusion

In summary, our study suggests that *LDHA* is abnormally downregulated, and *SLC16A1* is upregulated in LGG. Furthermore, these two genes have potential prognostic and diagnostic values for LGG. Importantly, low *SLC16A1* expression inhibited the proliferation, migration, and invasion of SW1088 and HS683 cells. *SLC16A1* may serve as a potential diagnostic and therapeutic target for LGG.

## Supplementary Information

Below is the link to the electronic supplementary material.

Supplementary Material 1.

Supplementary Material 2.

Supplementary Material 3.

## Data Availability

The datasets used or analysed during the current study are available from the corresponding author on reasonable request.
