# Supplementary material for: Identification and validation of LDHA and SLC16A1 for predicting prognosis and diagnosis in lower-grade glioma
Source: Discov Oncol. 2025 Aug 9;16:1511. doi: 10.1007/s12672-025-03297-2 (PMC12335421; doi:10.1007/s12672-025-03297-2)
Supplement: Supplementary file 1 — Supplementary Material 1. [file 12672_2025_3297_MOESM1_ESM.docx]

*
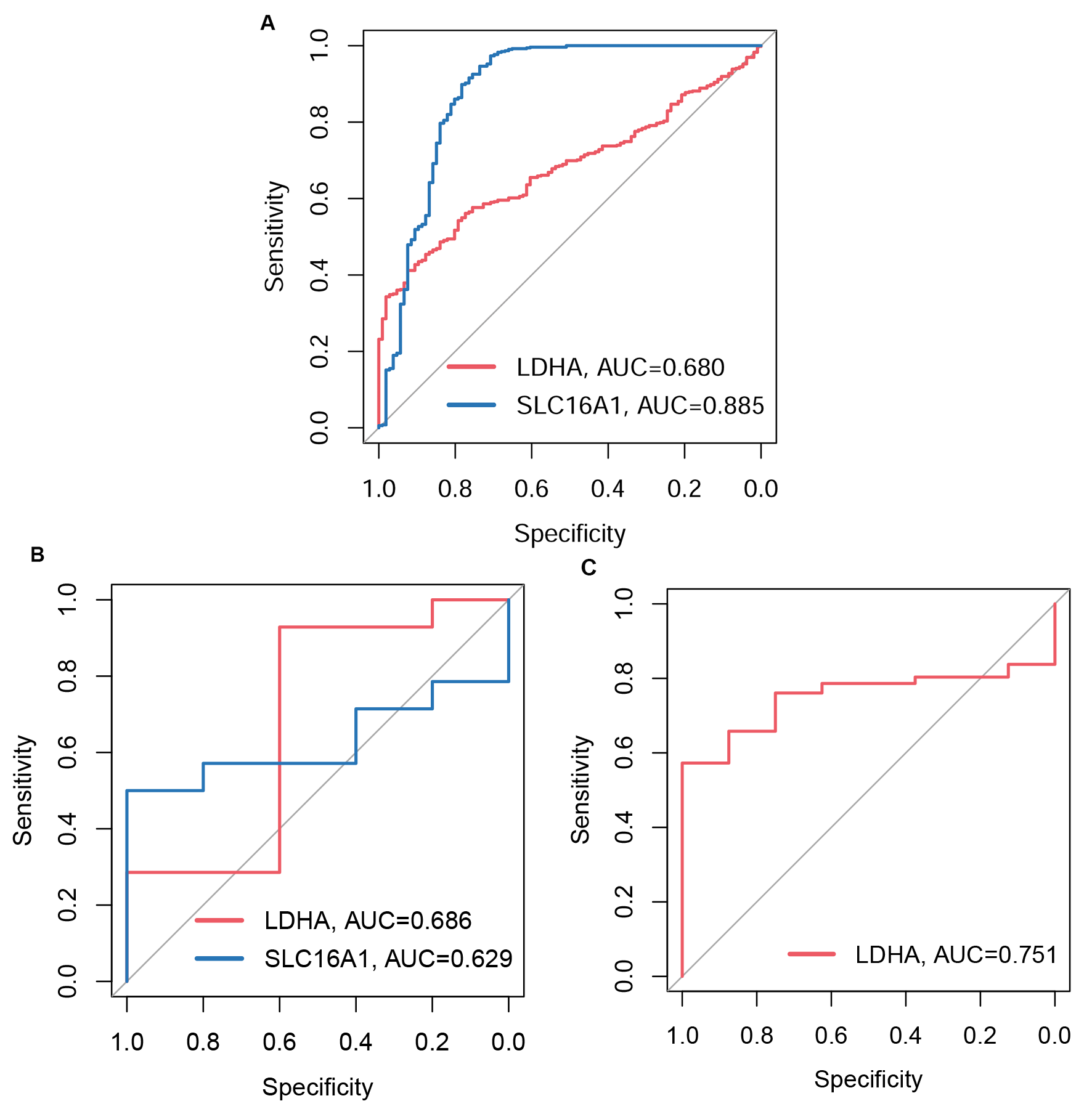
*

**Supplemental Fig 1** The diagnostic value of *LDHA* and *SLC16A1* by ROC curve analysis.

ROC curves of *LDHA* and *SLC16A1* in (A) TCGA-LGG, (B) GSE15824-LGG and (C) GSE16011-LGG. The abscissa represents specificity and the ordinate represents sensitivity.
