# Supplementary material for: Identification and validation of LDHA and SLC16A1 for predicting prognosis and diagnosis in lower-grade glioma
Source: Discov Oncol. 2025 Aug 9;16:1511. doi: 10.1007/s12672-025-03297-2 (PMC12335421; doi:10.1007/s12672-025-03297-2)
Supplement: Supplementary file 2 — Supplementary Material 2. [file 12672_2025_3297_MOESM2_ESM.docx]

**GSE15824 gene expression data**

| DATA | group | LDHA | SLC16A1 |
| --- | --- | --- | --- |
| GSM397626 | Tumor | 13.65912507 | 8.052234666 |
| GSM397627 | Tumor | 13.36429155 | 7.651197873 |
| GSM397628 | Tumor | 13.24672537 | 7.390343081 |
| GSM397629 | Tumor | 14.14031285 | 9.190644816 |
| GSM397630 | Tumor | 12.5369225 | 8.386251538 |
| GSM397631 | Tumor | 11.75315179 | 8.609607313 |
| GSM397632 | Tumor | 13.85939811 | 8.937698653 |
| GSM397633 | Tumor | 12.57362024 | 8.631225409 |
| GSM397634 | Tumor | 13.85456887 | 9.573015706 |
| GSM397635 | Tumor | 14.21847923 | 9.240878039 |
| GSM397636 | Tumor | 13.15515727 | 9.314047207 |
| GSM397637 | Tumor | 14.0342153 | 8.491775333 |
| GSM397638 | Tumor | 12.35477138 | 8.92549153 |
| GSM397639 | Tumor | 11.91567507 | 8.247572063 |
| GSM397640 | Tumor | 12.3732172 | 8.57629684 |
| GSM397641 | Tumor | 12.76525275 | 9.089962996 |
| GSM397642 | Tumor | 11.74955127 | 8.065824182 |
| GSM397643 | Tumor | 12.2507143 | 9.086862696 |
| GSM397644 | Tumor | 12.02499415 | 9.174627161 |
| GSM397645 | Tumor | 12.86420193 | 9.073181316 |
| GSM397646 | Tumor | 13.67341052 | 8.982308038 |
| GSM397647 | Tumor | 12.72627104 | 8.235988097 |
| GSM397648 | Tumor | 12.25061111 | 7.444654095 |
| GSM397649 | Tumor | 13.35766271 | 8.648873813 |
| GSM397650 | Tumor | 13.37986615 | 8.801019571 |
| GSM397651 | Tumor | 11.02888099 | 8.047863306 |
| GSM397652 | Tumor | 11.21579022 | 7.767218492 |
| GSM397653 | Tumor | 12.55793568 | 8.961118928 |
| GSM397654 | Tumor | 13.95143712 | 9.400941571 |
| GSM397655 | Tumor | 11.52162226 | 9.053183325 |
| GSM397656 | Normal | 13.50822872 | 8.777162625 |
| GSM397657 | Normal | 13.40780413 | 8.793035979 |
| GSM397658 | Normal | 13.80051578 | 9.064583233 |
| GSM397659 | Normal | 12.18947875 | 8.926697198 |
| GSM397660 | Normal | 12.15710131 | 8.597338731 |
| GSM397661 | Tumor | 13.29151607 | 8.235036311 |
| GSM397662 | Tumor | 13.39450517 | 8.205190856 |
| GSM397663 | Tumor | 13.16436444 | 8.744011646 |
| GSM397664 | Tumor | 13.16419796 | 8.76363236 |
| GSM397665 | Tumor | 13.26986712 | 8.533091455 |
| GSM397666 | Tumor | 13.29305015 | 8.248423353 |
| GSM397667 | Tumor | 12.89003614 | 8.971058819 |
| GSM397668 | Tumor | 12.96604049 | 8.932051948 |
| GSM397669 | Tumor | 13.22948876 | 9.35786224 |
| GSM397670 | Tumor | 13.15675601 | 9.367174657 |

**GSE16011 gene expression data**

| ID_REF | group | LDHA |
| --- | --- | --- |
| GSM405250 | Tumor | 10.02073493 |
| GSM405256 | Tumor | 12.11710034 |
| GSM405258 | Tumor | 11.27638111 |
| GSM405311 | Tumor | 11.86897511 |
| GSM405354 | Tumor | 12.57512782 |
| GSM405355 | Tumor | 13.27026604 |
| GSM405383 | Tumor | 12.90398126 |
| GSM405394 | Tumor | 10.85234854 |
| GSM405402 | Tumor | 11.98801514 |
| GSM405424 | Tumor | 11.83361931 |
| GSM405433 | Tumor | 9.760915051 |
| GSM405444 | Tumor | 10.58470276 |
| GSM405462 | Tumor | 11.24036385 |
| GSM405259 | Tumor | 12.50833352 |
| GSM405265 | Tumor | 12.26383593 |
| GSM405277 | Tumor | 11.67283869 |
| GSM405281 | Tumor | 12.32769931 |
| GSM405289 | Tumor | 12.34512915 |
| GSM405291 | Tumor | 12.58199823 |
| GSM405295 | Tumor | 12.24012696 |
| GSM405321 | Tumor | 12.34999366 |
| GSM405400 | Tumor | 12.41455611 |
| GSM405401 | Tumor | 10.94177079 |
| GSM405403 | Tumor | 12.63950557 |
| GSM405407 | Tumor | 12.60372707 |
| GSM405411 | Tumor | 11.77116137 |
| GSM405413 | Tumor | 9.559637819 |
| GSM405421 | Tumor | 11.16046375 |
| GSM405423 | Tumor | 11.23916502 |
| GSM405200 | Normal | 12.53861471 |
| GSM405209 | Normal | 11.72222886 |
| GSM405332 | Normal | 12.40680918 |
| GSM405336 | Normal | 12.60922591 |
| GSM405357 | Normal | 12.09221321 |
| GSM405358 | Normal | 12.48722477 |
| GSM405359 | Normal | 12.4887379 |
| GSM405360 | Normal | 12.55479 |
| GSM405213 | Tumor | 12.68027106 |
| GSM405214 | Tumor | 11.00261622 |
| GSM405215 | Tumor | 13.4068838 |
| GSM405216 | Tumor | 11.45872294 |
| GSM405217 | Tumor | 13.01650713 |
| GSM405218 | Tumor | 13.37279441 |
| GSM405219 | Tumor | 13.39199673 |
| GSM405220 | Tumor | 13.94257548 |
| GSM405221 | Tumor | 13.75141605 |
| GSM405222 | Tumor | 12.58425657 |
| GSM405223 | Tumor | 13.37245195 |
| GSM405224 | Tumor | 13.27408067 |
| GSM405228 | Tumor | 12.86891814 |
| GSM405229 | Tumor | 12.86787423 |
| GSM405230 | Tumor | 13.67914364 |
| GSM405231 | Tumor | 12.81554932 |
| GSM405232 | Tumor | 12.17420746 |
| GSM405233 | Tumor | 13.14294228 |
| GSM405234 | Tumor | 13.41746991 |
| GSM405235 | Tumor | 12.96358661 |
| GSM405236 | Tumor | 13.71677938 |
| GSM405237 | Tumor | 13.33513933 |
| GSM405238 | Tumor | 11.45558705 |
| GSM405239 | Tumor | 13.42603225 |
| GSM405240 | Tumor | 13.15068555 |
| GSM405241 | Tumor | 13.40199785 |
| GSM405242 | Tumor | 12.09210718 |
| GSM405243 | Tumor | 12.64666598 |
| GSM405244 | Tumor | 11.21836962 |
| GSM405245 | Tumor | 11.65078248 |
| GSM405246 | Tumor | 9.496469042 |
| GSM405247 | Tumor | 12.78716916 |
| GSM405248 | Tumor | 12.49825441 |
| GSM405249 | Tumor | 12.19748566 |
| GSM405251 | Tumor | 10.55249019 |
| GSM405252 | Tumor | 13.70185695 |
| GSM405253 | Tumor | 12.48995477 |
| GSM405254 | Tumor | 13.1940596 |
| GSM405255 | Tumor | 12.87246599 |
| GSM405260 | Tumor | 13.55208083 |
| GSM405262 | Tumor | 13.91242233 |
| GSM405263 | Tumor | 13.07745956 |
| GSM405264 | Tumor | 12.95405438 |
| GSM405266 | Tumor | 13.48188108 |
| GSM405267 | Tumor | 11.96696509 |
| GSM405268 | Tumor | 12.88785059 |
| GSM405269 | Tumor | 13.5050477 |
| GSM405270 | Tumor | 12.92383417 |
| GSM405271 | Tumor | 13.90542206 |
| GSM405274 | Tumor | 13.62894687 |
| GSM405275 | Tumor | 12.28259125 |
| GSM405276 | Tumor | 13.5459175 |
| GSM405278 | Tumor | 12.25520104 |
| GSM405280 | Tumor | 12.65925281 |
| GSM405282 | Tumor | 13.48251357 |
| GSM405290 | Tumor | 12.90737556 |
| GSM405292 | Tumor | 12.468942 |
| GSM405293 | Tumor | 13.46941459 |
| GSM405294 | Tumor | 13.00568624 |
| GSM405296 | Tumor | 13.0733654 |
| GSM405297 | Tumor | 12.78057293 |
| GSM405299 | Tumor | 13.39270557 |
| GSM405301 | Tumor | 13.65241691 |
| GSM405302 | Tumor | 13.46996401 |
| GSM405303 | Tumor | 12.81829587 |
| GSM405304 | Tumor | 12.74580463 |
| GSM405305 | Tumor | 13.33085854 |
| GSM405307 | Tumor | 13.38424531 |
| GSM405308 | Tumor | 10.32788698 |
| GSM405309 | Tumor | 12.94840925 |
| GSM405312 | Tumor | 13.46596484 |
| GSM405313 | Tumor | 12.22799926 |
| GSM405314 | Tumor | 13.51985812 |
| GSM405315 | Tumor | 12.2048537 |
| GSM405317 | Tumor | 13.68342852 |
| GSM405320 | Tumor | 13.00430473 |
| GSM405322 | Tumor | 13.58856273 |
| GSM405323 | Tumor | 12.99964145 |
| GSM405324 | Tumor | 13.68219919 |
| GSM405326 | Tumor | 13.49317539 |
| GSM405328 | Tumor | 13.33791857 |
| GSM405330 | Tumor | 12.51333156 |
| GSM405337 | Tumor | 12.75390243 |
| GSM405339 | Tumor | 13.69879345 |
| GSM405340 | Tumor | 12.4453985 |
| GSM405343 | Tumor | 13.80776571 |
| GSM405345 | Tumor | 12.29598994 |
| GSM405349 | Tumor | 12.04733717 |
| GSM405350 | Tumor | 11.59788531 |
| GSM405351 | Tumor | 11.88001617 |
| GSM405352 | Tumor | 12.44176013 |
| GSM405353 | Tumor | 13.40745126 |
| GSM405356 | Tumor | 11.47446395 |
| GSM405362 | Tumor | 13.69202223 |
| GSM405363 | Tumor | 11.98950704 |
| GSM405365 | Tumor | 11.95554496 |
| GSM405367 | Tumor | 13.13906742 |
| GSM405368 | Tumor | 12.72879003 |
| GSM405369 | Tumor | 10.15005053 |
| GSM405370 | Tumor | 11.42015981 |
| GSM405371 | Tumor | 12.77751284 |
| GSM405372 | Tumor | 12.75912017 |
| GSM405373 | Tumor | 13.4538376 |
| GSM405374 | Tumor | 13.68490022 |
| GSM405375 | Tumor | 13.8413433 |
| GSM405376 | Tumor | 12.57188268 |
| GSM405379 | Tumor | 12.52073061 |
| GSM405384 | Tumor | 13.83682104 |
| GSM405385 | Tumor | 10.6439249 |
| GSM405389 | Tumor | 11.34597153 |
| GSM405391 | Tumor | 13.64747298 |
| GSM405392 | Tumor | 13.3241105 |
| GSM405393 | Tumor | 13.47567802 |
| GSM405396 | Tumor | 13.00076097 |
| GSM405397 | Tumor | 12.94286527 |
| GSM405398 | Tumor | 13.42710491 |
| GSM405405 | Tumor | 12.89532195 |
| GSM405412 | Tumor | 11.30067652 |
| GSM405415 | Tumor | 13.85572627 |
| GSM405416 | Tumor | 13.06068434 |
| GSM405417 | Tumor | 12.37638819 |
| GSM405418 | Tumor | 10.25053627 |
| GSM405419 | Tumor | 13.03633779 |
| GSM405422 | Tumor | 12.33282134 |
| GSM405426 | Tumor | 13.06382629 |
| GSM405427 | Tumor | 12.43611492 |
| GSM405428 | Tumor | 13.54430972 |
| GSM405430 | Tumor | 13.12920683 |
| GSM405431 | Tumor | 12.544065 |
| GSM405432 | Tumor | 13.52398905 |
| GSM405434 | Tumor | 13.53522303 |
| GSM405436 | Tumor | 13.29382704 |
| GSM405438 | Tumor | 13.65826407 |
| GSM405440 | Tumor | 13.5517182 |
| GSM405442 | Tumor | 13.46944879 |
| GSM405443 | Tumor | 13.53382135 |
| GSM405446 | Tumor | 13.4763447 |
| GSM405447 | Tumor | 13.44517469 |
| GSM405448 | Tumor | 13.18422335 |
| GSM405452 | Tumor | 13.34287325 |
| GSM405453 | Tumor | 13.63348559 |
| GSM405454 | Tumor | 13.27095871 |
| GSM405455 | Tumor | 12.27493655 |
| GSM405456 | Tumor | 13.51722126 |
| GSM405458 | Tumor | 11.59445486 |
| GSM405459 | Tumor | 12.94170529 |
| GSM405461 | Tumor | 12.44169085 |
| GSM405463 | Tumor | 13.54161067 |
| GSM405464 | Tumor | 12.30812484 |
| GSM405465 | Tumor | 13.30779761 |
| GSM405466 | Tumor | 13.55262468 |
| GSM405470 | Tumor | 12.3533135 |
| GSM405471 | Tumor | 13.7270012 |
| GSM405472 | Tumor | 13.46876153 |
| GSM405473 | Tumor | 12.70135822 |
| GSM405474 | Tumor | 13.08275417 |
| GSM405475 | Tumor | 10.76110011 |
| GSM405477 | Tumor | 13.04967494 |
| GSM405479 | Tumor | 12.92834498 |
| GSM405395 | Tumor | 10.23790995 |
| GSM405439 | Tumor | 11.01876611 |
| GSM405451 | Tumor | 11.6393131 |
| GSM405225 | Tumor | 9.89866242 |
| GSM405226 | Tumor | 13.31715918 |
| GSM405273 | Tumor | 12.71402842 |
| GSM405279 | Tumor | 13.28772997 |
| GSM405283 | Tumor | 10.59541794 |
| GSM405284 | Tumor | 12.07847602 |
| GSM405285 | Tumor | 12.82541899 |
| GSM405286 | Tumor | 11.11415751 |
| GSM405288 | Tumor | 11.84290047 |
| GSM405298 | Tumor | 10.26327566 |
| GSM405300 | Tumor | 12.36513124 |
| GSM405310 | Tumor | 10.27342578 |
| GSM405316 | Tumor | 12.21036341 |
| GSM405325 | Tumor | 12.34269729 |
| GSM405341 | Tumor | 13.34513436 |
| GSM405347 | Tumor | 12.45136199 |
| GSM405382 | Tumor | 10.26374289 |
| GSM405387 | Tumor | 11.52165711 |
| GSM405388 | Tumor | 13.36533244 |
| GSM405390 | Tumor | 12.57112032 |
| GSM405399 | Tumor | 12.71003843 |
| GSM405429 | Tumor | 9.324175605 |
| GSM405435 | Tumor | 11.68729675 |
| GSM405450 | Tumor | 11.9211271 |
| GSM405476 | Tumor | 12.47622571 |
| GSM405338 | Tumor | 9.962006302 |
| GSM405344 | Tumor | 12.24958997 |
| GSM405378 | Tumor | 12.33250937 |
| GSM405386 | Tumor | 11.5590399 |
| GSM405408 | Tumor | 10.28579111 |
| GSM405425 | Tumor | 9.782396862 |
| GSM405437 | Tumor | 9.888219896 |
| GSM405441 | Tumor | 9.722734484 |
| GSM405201 | Tumor | 10.21050691 |
| GSM405202 | Tumor | 11.26282331 |
| GSM405203 | Tumor | 11.33852102 |
| GSM405204 | Tumor | 10.6881794 |
| GSM405205 | Tumor | 11.59916507 |
| GSM405206 | Tumor | 10.12318131 |
| GSM405207 | Tumor | 10.03374089 |
| GSM405208 | Tumor | 10.25797302 |
| GSM405210 | Tumor | 10.01860158 |
| GSM405211 | Tumor | 11.00270911 |
| GSM405212 | Tumor | 10.16484156 |
| GSM405227 | Tumor | 10.37763323 |
| GSM405257 | Tumor | 10.35365762 |
| GSM405261 | Tumor | 13.3817558 |
| GSM405272 | Tumor | 12.68614314 |
| GSM405287 | Tumor | 10.58471789 |
| GSM405318 | Tumor | 10.34390999 |
| GSM405319 | Tumor | 10.62161558 |
| GSM405327 | Tumor | 10.36537599 |
| GSM405329 | Tumor | 10.41440643 |
| GSM405331 | Tumor | 10.07577986 |
| GSM405333 | Tumor | 11.16227503 |
| GSM405334 | Tumor | 13.02032216 |
| GSM405335 | Tumor | 9.06534827 |
| GSM405342 | Tumor | 11.32942238 |
| GSM405361 | Tumor | 12.29655152 |
| GSM405364 | Tumor | 13.05200677 |
| GSM405366 | Tumor | 10.82226663 |
| GSM405377 | Tumor | 12.81970032 |
| GSM405380 | Tumor | 10.80543956 |
| GSM405381 | Tumor | 10.82977899 |
| GSM405404 | Tumor | 12.68159326 |
| GSM405406 | Tumor | 9.659886638 |
| GSM405409 | Tumor | 10.57957233 |
| GSM405410 | Tumor | 11.78168984 |
| GSM405414 | Tumor | 11.65636039 |
| GSM405420 | Tumor | 9.894538905 |
| GSM405445 | Tumor | 12.76746258 |
| GSM405449 | Tumor | 10.77408408 |
| GSM405457 | Tumor | 12.93214354 |
| GSM405460 | Tumor | 13.29119905 |
| GSM405467 | Tumor | 9.910615621 |
| GSM405469 | Tumor | 9.498740156 |
| GSM405478 | Tumor | 10.74089127 |
| GSM405306 | Tumor | 12.0183688 |
| GSM405346 | Tumor | 11.63507567 |
| GSM405348 | Tumor | 11.71635372 |
| GSM405468 | Tumor | 12.53717647 |
| GSM405480 | Tumor | 11.30800606 |
| GSM405481 | Tumor | 11.74856081 |
| GSM405482 | Tumor | 10.87823422 |
| GSM405483 | Tumor | 11.39305795 |

**TCGA gene expression data**

| ID | group | LDHA | SLC16A1 |
| --- | --- | --- | --- |
| GTEX-1117F-3226-SM-5N9CT | Normal | 0.606800425 | 0.534371579 |
| GTEX-111FC-3126-SM-5GZZ2 | Normal | 0.918039463 | 0.386719155 |
| GTEX-1128S-2726-SM-5H12C | Normal | 0.895359922 | 0.479187228 |
| GTEX-117XS-3026-SM-5N9CA | Normal | 0.94841073 | 0.352332356 |
| GTEX-1192X-3126-SM-5N9BY | Normal | 0.965477612 | 0.578742297 |
| GTEX-11DXW-1126-SM-5H12Q | Normal | 0.851789566 | 0.279139605 |
| GTEX-11DXY-3226-SM-5GIDE | Normal | 1.03588437 | 0.368933131 |
| GTEX-11EI6-3026-SM-5GZZO | Normal | 0.71321511 | 0.396378904 |
| GTEX-11EMC-3226-SM-5EGKW | Normal | 0.735996876 | 0.75270755 |
| GTEX-11GS4-3126-SM-5A5LH | Normal | 0.853297406 | 0.403968533 |
| GTEX-11GSO-2926-SM-5HL73 | Normal | 0.876267695 | 0.632683378 |
| GTEX-11GSP-3226-SM-5986O | Normal | 0.660845844 | 0.398403866 |
| GTEX-11NUK-2926-SM-5A5MD | Normal | 0.903181594 | 0.387377632 |
| GTEX-11NV4-2126-SM-5N9DS | Normal | 0.693534051 | 0.260152702 |
| GTEX-11O72-2926-SM-5BC4V | Normal | 0.948909815 | 0.50956866 |
| GTEX-11ONC-2926-SM-5P9JM | Normal | 0.890750565 | 0.482789788 |
| GTEX-11TTK-2926-SM-5PNYP | Normal | 0.933975216 | 0.516463826 |
| GTEX-11TUW-3126-SM-5GU4Y | Normal | 1.097630659 | 0.465234067 |
| GTEX-11WQK-3026-SM-5EQL6 | Normal | 1.082793768 | 0.420155624 |
| GTEX-11ZUS-2926-SM-5FQSL | Normal | 0.948902815 | 0.327307474 |
| GTEX-12126-1026-SM-5P9JJ | Normal | 0.815055607 | 0.372650092 |
| GTEX-12696-2926-SM-5FQTG | Normal | 0.836347504 | 0.708393022 |
| GTEX-12WSA-2926-SM-5EQ4D | Normal | 0.668599334 | 0.663317109 |
| GTEX-12WSC-3026-SM-5GCNF | Normal | 0.89354314 | 0.334972893 |
| GTEX-12WSD-3126-SM-5HL7P | Normal | 0.71552407 | 0.379989535 |
| GTEX-12WSH-3026-SM-5CVNI | Normal | 0.760644081 | 0.493897195 |
| GTEX-12ZZW-2926-SM-5LZUP | Normal | 0.799181578 | 0.600412826 |
| GTEX-12ZZX-2926-SM-5GCOQ | Normal | 0.895635067 | 0.35531463 |
| GTEX-12ZZY-3026-SM-5GCOU | Normal | 0.855051255 | 0.441307817 |
| GTEX-12ZZZ-3026-SM-5BC67 | Normal | 0.858847275 | 0.478046295 |
| GTEX-1313W-3126-SM-5LZUI | Normal | 0.925721373 | 0.364971917 |
| GTEX-131YS-3126-SM-5KLYT | Normal | 0.955129767 | 0.349226465 |
| GTEX-132Q8-3026-SM-5PNVG | Normal | 1.071152194 | 0.569866334 |
| GTEX-1399T-3026-SM-5KLZC | Normal | 0.820973121 | 0.409434129 |
| GTEX-139UC-3126-SM-5J1OY | Normal | 0.848488064 | 0.411618891 |
| GTEX-13FHO-3026-SM-5J1O9 | Normal | 1.111806906 | 0.579937209 |
| GTEX-13FHP-3026-SM-5IJBS | Normal | 0.844742932 | 0.477961039 |
| GTEX-13FLW-1426-SM-5K7YE | Normal | 0.970878198 | 0.393923804 |
| GTEX-13G51-3026-SM-5IJB8 | Normal | 0.739268851 | 0.610724688 |
| GTEX-13IVO-2926-SM-5L3CZ | Normal | 0.897945879 | 0.835774117 |
| GTEX-13NYB-3026-SM-5IJD7 | Normal | 0.846601742 | 0.468255423 |
| GTEX-13NYC-2826-SM-5K7WR | Normal | 1.313874404 | 0.54680615 |
| GTEX-13NYS-3126-SM-5KLYV | Normal | 1.059149315 | 0.491590353 |
| GTEX-13O3O-3126-SM-5KM3H | Normal | 0.950629481 | 0.49548017 |
| GTEX-13O3Q-2926-SM-5KM45 | Normal | 0.934233006 | 0.388384798 |
| GTEX-13OVH-3026-SM-5MR4N | Normal | 1.068150911 | 0.410903645 |
| GTEX-13OVJ-2826-SM-5L3GW | Normal | 1.07720887 | 0.563233436 |
| GTEX-13OW6-3026-SM-5J2MI | Normal | 0.918059635 | 0.473835507 |
| GTEX-13OW7-3026-SM-5L3GY | Normal | 0.680317964 | 0.378352529 |
| GTEX-13PL6-3126-SM-5LUAR | Normal | 0.832368274 | 0.45930555 |
| GTEX-13QIC-2926-SM-5J2NF | Normal | 0.822149825 | 0.591462738 |
| GTEX-13S7M-3126-SM-5RQJQ | Normal | 0.756072746 | 0.83378032 |
| GTEX-13SLX-3126-SM-5S2Q5 | Normal | 0.919632802 | 0.419030067 |
| GTEX-13VXU-2926-SM-5LU5C | Normal | 0.999046956 | 0.300792057 |
| GTEX-13X6J-3026-SM-5Q5CU | Normal | 0.836757262 | 0.31415708 |
| GTEX-1445S-3026-SM-5O9BR | Normal | 0.785724523 | 0.616857527 |
| GTEX-144FL-3026-SM-5O99C | Normal | 0.861870912 | 0.475092541 |
| GTEX-144GL-3026-SM-5Q5CW | Normal | 0.773844532 | 0.285384568 |
| GTEX-145LS-3126-SM-5Q5BY | Normal | 0.948019369 | 0.684440545 |
| GTEX-145MF-2726-SM-5O995 | Normal | 0.928164815 | 0.368461693 |
| GTEX-145MG-3026-SM-5RQJA | Normal | 0.831951432 | 0.306754877 |
| GTEX-145MH-3026-SM-5Q5DZ | Normal | 1.074599577 | 0.388882709 |
| GTEX-14A5I-2926-SM-5Q5CQ | Normal | 0.769241912 | 0.420427595 |
| GTEX-14ASI-3026-SM-5S2PN | Normal | 0.980885195 | 0.399025785 |
| GTEX-14BMV-3026-SM-5S2PQ | Normal | 1.037037341 | 0.311663957 |
| GTEX-N7MS-2425-SM-26GMD | Normal | 0.555435157 | 0.614853226 |
| GTEX-N7MT-1126-SM-2YUNQ | Normal | 0.664024501 | 0.491949379 |
| GTEX-NPJ7-2726-SM-2I3FT | Normal | 0.952630507 | 0.80259198 |
| GTEX-NPJ8-1526-SM-26GMH | Normal | 0.782241815 | 0.694851961 |
| GTEX-NPJ8-1526-SM-26GMY | Normal | 1.233105289 | 1.004582775 |
| GTEX-NPJ8-1526-SM-2D7VU | Normal | 0.918050877 | 0.693486036 |
| GTEX-PVOW-2526-SM-2XCF7 | Normal | 1.143019796 | 0.394756981 |
| GTEX-Q2AG-2926-SM-2HMJ3 | Normal | 0.909610061 | 0.563474336 |
| GTEX-QDT8-2926-SM-32PKC | Normal | 0.809563067 | 1.031181943 |
| GTEX-QVUS-2826-SM-3GADB | Normal | 0.83119777 | 0.689714733 |
| GTEX-R55F-1326-SM-2TF5F | Normal | 1.020083767 | 0.329220222 |
| GTEX-R55F-1326-SM-5S2V4 | Normal | 1.132140912 | 0.37959642 |
| GTEX-RNOR-2326-SM-2TF4I | Normal | 0.954190651 | 0.522885273 |
| GTEX-RU72-3026-SM-5SI7Y | Normal | 0.785753957 | 0.489914139 |
| GTEX-T2IS-3026-SM-32QPM | Normal | 1.082106533 | 0.589202177 |
| GTEX-T5JC-2426-SM-3NMDB | Normal | 1.134468288 | 0.399862879 |
| GTEX-T6MN-2626-SM-32PMQ | Normal | 1.000326957 | 0.337715904 |
| GTEX-TSE9-3026-SM-3DB76 | Normal | 1.089592001 | 0.448389093 |
| GTEX-WHSE-3026-SM-3P5ZH | Normal | 1.196742119 | 0.391255367 |
| GTEX-WL46-2926-SM-3LK82 | Normal | 1.043571185 | 0.400056233 |
| GTEX-WVLH-3026-SM-3MJG9 | Normal | 0.992967188 | 0.367624549 |
| GTEX-WWYW-3126-SM-3NB39 | Normal | 1.293977559 | 0.430622192 |
| GTEX-WZTO-2926-SM-3NM9I | Normal | 1.168354603 | 0.332108196 |
| GTEX-X4XX-3026-SM-3NMB2 | Normal | 0.890233824 | 0.475773368 |
| GTEX-X585-3026-SM-46MWF | Normal | 1.68866983 | 0.480420601 |
| GTEX-XLM4-3026-SM-4AT6L | Normal | 0.989484969 | 0.550781993 |
| GTEX-XMD1-2926-SM-4AT42 | Normal | 1.357360797 | 0.7034874 |
| GTEX-XOTO-3026-SM-4B65M | Normal | 1.002663359 | 0.356916024 |
| GTEX-Y8DK-0826-SM-4TT3T | Normal | 0.935731236 | 0.331724573 |
| GTEX-Z93S-2926-SM-57WB9 | Normal | 0.847525953 | 0.364345893 |
| GTEX-ZAJG-3126-SM-5HL9J | Normal | 0.731511892 | 0.246511809 |
| GTEX-ZAK1-3026-SM-5S2MJ | Normal | 0.912470321 | 0.537107868 |
| GTEX-ZE7O-3126-SM-5HL5X | Normal | 0.682338343 | 0.326087893 |
| GTEX-ZE9C-3026-SM-4WKHB | Normal | 1.093429902 | 0.367553296 |
| GTEX-ZF28-3026-SM-4WKHP | Normal | 0.839356835 | 0.552073141 |
| GTEX-ZUA1-3026-SM-59HJC | Normal | 0.960511423 | 0.423092146 |
| GTEX-ZVT3-3026-SM-5E43N | Normal | 0.78441755 | 0.649129284 |
| GTEX-ZYFD-3026-SM-5E44C | Normal | 0.987617939 | 0.337545302 |
| GTEX-ZYY3-3126-SM-5SI9L | Normal | 1.183604443 | 0.740798158 |
| GTEX-ZZPT-3026-SM-5GZXH | Normal | 0.910695217 | 0.375822332 |
| TCGA-CS-4938-01 | Tumor | 0.893560442 | 0.662529299 |
| TCGA-CS-4941-01 | Tumor | 1.067747382 | 0.859484808 |
| TCGA-CS-4942-01 | Tumor | 0.835684097 | 0.720113057 |
| TCGA-CS-4943-01 | Tumor | 0.497216193 | 0.720527076 |
| TCGA-CS-4944-01 | Tumor | 0.913635926 | 0.630695994 |
| TCGA-CS-5390-01 | Tumor | 0.521803875 | 0.634061125 |
| TCGA-CS-5393-01 | Tumor | 0.532289741 | 0.810634104 |
| TCGA-CS-5394-01 | Tumor | 0.325960645 | 0.810026442 |
| TCGA-CS-5395-01 | Tumor | 0.957097655 | 0.984342751 |
| TCGA-CS-5396-01 | Tumor | 0.67305873 | 0.591221847 |
| TCGA-CS-5397-01 | Tumor | 1.070768341 | 0.782288281 |
| TCGA-CS-6186-01 | Tumor | 0.899537158 | 0.777466006 |
| TCGA-CS-6188-01 | Tumor | 1.216169031 | 0.911708963 |
| TCGA-CS-6290-01 | Tumor | 0.808277753 | 0.729633924 |
| TCGA-CS-6666-01 | Tumor | 1.206309766 | 0.843882713 |
| TCGA-CS-6667-01 | Tumor | 0.702350884 | 0.943246918 |
| TCGA-CS-6668-01 | Tumor | 0.433299992 | 0.602678767 |
| TCGA-CS-6669-01 | Tumor | 1.159369124 | 0.62139168 |
| TCGA-CS-6670-01 | Tumor | 0.557619961 | 0.613154983 |
| TCGA-DB-5270-01 | Tumor | 0.927006729 | 0.642532468 |
| TCGA-DB-5273-01 | Tumor | 0.80054827 | 0.65057128 |
| TCGA-DB-5274-01 | Tumor | 0.330271188 | 0.66586049 |
| TCGA-DB-5275-01 | Tumor | 0.754315607 | 0.703885549 |
| TCGA-DB-5276-01 | Tumor | 0.995686493 | 0.731158019 |
| TCGA-DB-5277-01 | Tumor | 0.528817531 | 0.697827032 |
| TCGA-DB-5278-01 | Tumor | 0.716074335 | 0.547280598 |
| TCGA-DB-5279-01 | Tumor | 0.619305699 | 0.735159509 |
| TCGA-DB-5280-01 | Tumor | 0.875674298 | 0.976176528 |
| TCGA-DB-5281-01 | Tumor | 1.134777236 | 0.795230302 |
| TCGA-DB-A4X9-01 | Tumor | 0.818825443 | 0.694217458 |
| TCGA-DB-A4XA-01 | Tumor | 0.488974514 | 0.64212074 |
| TCGA-DB-A4XB-01 | Tumor | 1.012480407 | 0.644016232 |
| TCGA-DB-A4XC-01 | Tumor | 0.712040246 | 0.612487016 |
| TCGA-DB-A4XD-01 | Tumor | 0.885945402 | 0.771866873 |
| TCGA-DB-A4XE-01 | Tumor | 0.832966418 | 0.675776946 |
| TCGA-DB-A4XF-01 | Tumor | 0.815571477 | 0.679391666 |
| TCGA-DB-A4XG-01 | Tumor | 0.494120366 | 0.762109039 |
| TCGA-DB-A4XH-01 | Tumor | 0.47799798 | 0.618886452 |
| TCGA-DB-A64L-01 | Tumor | 0.762988275 | 0.677678845 |
| TCGA-DB-A64O-01 | Tumor | 0.88142738 | 0.667272846 |
| TCGA-DB-A64P-01 | Tumor | 0.513426832 | 0.673619123 |
| TCGA-DB-A64Q-01 | Tumor | 0.390521132 | 0.642661467 |
| TCGA-DB-A64R-01 | Tumor | 0.610663854 | 0.580250295 |
| TCGA-DB-A64S-01 | Tumor | 1.029742026 | 0.701383725 |
| TCGA-DB-A64U-01 | Tumor | 0.415497199 | 0.647194671 |
| TCGA-DB-A64V-01 | Tumor | 0.501167253 | 0.575636609 |
| TCGA-DB-A64W-01 | Tumor | 0.826522405 | 0.720426061 |
| TCGA-DB-A64X-01 | Tumor | 0.384369897 | 0.658237149 |
| TCGA-DB-A75K-01 | Tumor | 0.7482555 | 0.571930779 |
| TCGA-DB-A75L-01 | Tumor | 0.988908351 | 0.718176296 |
| TCGA-DB-A75M-01 | Tumor | 0.663106338 | 0.640211666 |
| TCGA-DB-A75O-01 | Tumor | 0.925541502 | 0.729421414 |
| TCGA-DB-A75P-01 | Tumor | 0.700121721 | 0.457147082 |
| TCGA-DH-5140-01 | Tumor | 0.868124105 | 0.791067505 |
| TCGA-DH-5141-01 | Tumor | 0.47682111 | 0.693063728 |
| TCGA-DH-5142-01 | Tumor | 0.870009181 | 0.764494507 |
| TCGA-DH-5143-01 | Tumor | 0.963679304 | 0.679837326 |
| TCGA-DH-5144-01 | Tumor | 0.482711383 | 0.605979979 |
| TCGA-DH-A669-01 | Tumor | 0.411878334 | 0.731471229 |
| TCGA-DH-A669-02 | Tumor | 0.444244228 | 0.68285404 |
| TCGA-DH-A66B-01 | Tumor | 1.024420914 | 0.728717216 |
| TCGA-DH-A66D-01 | Tumor | 0.484788993 | 0.698543549 |
| TCGA-DH-A66F-01 | Tumor | 0.69012524 | 0.718768943 |
| TCGA-DH-A7UR-01 | Tumor | 0.514878561 | 0.623971566 |
| TCGA-DH-A7US-01 | Tumor | 0.454513788 | 0.579077834 |
| TCGA-DH-A7UT-01 | Tumor | 0.581844597 | 0.648654226 |
| TCGA-DH-A7UU-01 | Tumor | 0.792985253 | 0.89090596 |
| TCGA-DH-A7UV-01 | Tumor | 0.888194225 | 0.773302254 |
| TCGA-DU-5847-01 | Tumor | 1.340180338 | 0.783928958 |
| TCGA-DU-5849-01 | Tumor | 0.702315087 | 0.619542168 |
| TCGA-DU-5852-01 | Tumor | 1.093244115 | 0.898424644 |
| TCGA-DU-5853-01 | Tumor | 0.849792224 | 0.658619236 |
| TCGA-DU-5854-01 | Tumor | 1.013361518 | 0.734492912 |
| TCGA-DU-5855-01 | Tumor | 0.807893724 | 0.781281574 |
| TCGA-DU-5870-01 | Tumor | 0.428718445 | 0.684506046 |
| TCGA-DU-5870-02 | Tumor | 0.337070429 | 0.724793212 |
| TCGA-DU-5871-01 | Tumor | 0.800506546 | 0.722593949 |
| TCGA-DU-5872-01 | Tumor | 0.760511671 | 0.859972075 |
| TCGA-DU-5872-02 | Tumor | 0.983226905 | 0.581188534 |
| TCGA-DU-5874-01 | Tumor | 0.434836982 | 0.605012849 |
| TCGA-DU-6392-01 | Tumor | 1.265260053 | 0.743745128 |
| TCGA-DU-6393-01 | Tumor | 0.44351213 | 0.711335331 |
| TCGA-DU-6394-01 | Tumor | 0.389822586 | 0.771146082 |
| TCGA-DU-6395-01 | Tumor | 0.448587851 | 0.617975501 |
| TCGA-DU-6396-01 | Tumor | 0.964057972 | 0.716936798 |
| TCGA-DU-6397-02 | Tumor | 0.510999679 | 0.660828478 |
| TCGA-DU-6399-01 | Tumor | 0.789107817 | 0.781368647 |
| TCGA-DU-6400-01 | Tumor | 0.70481489 | 0.688507587 |
| TCGA-DU-6401-01 | Tumor | 0.89655644 | 0.798797057 |
| TCGA-DU-6402-01 | Tumor | 1.386457437 | 0.732792971 |
| TCGA-DU-6403-01 | Tumor | 1.009022962 | 0.775936505 |
| TCGA-DU-6404-01 | Tumor | 1.24076871 | 0.888305955 |
| TCGA-DU-6404-02 | Tumor | 1.334517378 | 0.837211503 |
| TCGA-DU-6405-01 | Tumor | 1.167092327 | 0.788377568 |
| TCGA-DU-6406-01 | Tumor | 1.15112614 | 0.769961257 |
| TCGA-DU-6407-01 | Tumor | 0.409852809 | 0.724732746 |
| TCGA-DU-6407-02 | Tumor | 0.692873662 | 0.803124222 |
| TCGA-DU-6408-01 | Tumor | 0.800211384 | 0.648253417 |
| TCGA-DU-6410-01 | Tumor | 0.507667768 | 0.68554045 |
| TCGA-DU-6542-01 | Tumor | 1.086401816 | 0.747572474 |
| TCGA-DU-7006-01 | Tumor | 1.256081655 | 0.709584548 |
| TCGA-DU-7007-01 | Tumor | 0.943982746 | 0.961203817 |
| TCGA-DU-7008-01 | Tumor | 0.964389847 | 0.706008305 |
| TCGA-DU-7009-01 | Tumor | 0.637674508 | 0.674577044 |
| TCGA-DU-7010-01 | Tumor | 0.597109873 | 0.957384217 |
| TCGA-DU-7011-01 | Tumor | 0.914459957 | 0.747459537 |
| TCGA-DU-7012-01 | Tumor | 1.046582328 | 0.835040913 |
| TCGA-DU-7013-01 | Tumor | 0.980645086 | 0.880937371 |
| TCGA-DU-7014-01 | Tumor | 0.848872529 | 0.610827377 |
| TCGA-DU-7015-01 | Tumor | 0.884296188 | 0.737728474 |
| TCGA-DU-7018-01 | Tumor | 0.436313457 | 0.626255819 |
| TCGA-DU-7019-01 | Tumor | 0.605274016 | 0.81825875 |
| TCGA-DU-7290-01 | Tumor | 1.060425562 | 1.152696023 |
| TCGA-DU-7292-01 | Tumor | 1.091037139 | 0.780017254 |
| TCGA-DU-7294-01 | Tumor | 0.550690672 | 0.646422289 |
| TCGA-DU-7298-01 | Tumor | 0.7027683 | 0.857688399 |
| TCGA-DU-7299-01 | Tumor | 0.910188241 | 0.754298461 |
| TCGA-DU-7300-01 | Tumor | 0.870312411 | 0.641729807 |
| TCGA-DU-7301-01 | Tumor | 0.606175243 | 0.814999579 |
| TCGA-DU-7302-01 | Tumor | 0.883895624 | 0.6593 |
| TCGA-DU-7304-02 | Tumor | 0.287032715 | 0.521209831 |
| TCGA-DU-7306-01 | Tumor | 0.416381463 | 0.86146899 |
| TCGA-DU-7309-01 | Tumor | 0.923859021 | 0.670820103 |
| TCGA-DU-8158-01 | Tumor | 1.04873943 | 0.93283074 |
| TCGA-DU-8161-01 | Tumor | 1.088607302 | 0.789482259 |
| TCGA-DU-8162-01 | Tumor | 1.130745183 | 0.704265703 |
| TCGA-DU-8163-01 | Tumor | 0.71870018 | 0.795291763 |
| TCGA-DU-8164-01 | Tumor | 0.579848408 | 0.579429614 |
| TCGA-DU-8165-01 | Tumor | 1.108029415 | 1.139312888 |
| TCGA-DU-8166-01 | Tumor | 0.821711757 | 0.662194013 |
| TCGA-DU-8167-01 | Tumor | 0.626454897 | 0.703235726 |
| TCGA-DU-8168-01 | Tumor | 0.58008157 | 0.763692102 |
| TCGA-DU-A5TP-01 | Tumor | 1.513092959 | 0.668339955 |
| TCGA-DU-A5TR-01 | Tumor | 0.532885502 | 0.57015266 |
| TCGA-DU-A5TS-01 | Tumor | 0.54518555 | 0.588941465 |
| TCGA-DU-A5TT-01 | Tumor | 0.712139507 | 0.764239563 |
| TCGA-DU-A5TU-01 | Tumor | 0.730306391 | 0.651884416 |
| TCGA-DU-A5TW-01 | Tumor | 0.705068172 | 0.838496173 |
| TCGA-DU-A5TY-01 | Tumor | 1.011484506 | 0.802092226 |
| TCGA-DU-A6S2-01 | Tumor | 0.902999216 | 0.738865407 |
| TCGA-DU-A6S3-01 | Tumor | 0.581179198 | 0.582851002 |
| TCGA-DU-A6S6-01 | Tumor | 0.821078891 | 0.494555938 |
| TCGA-DU-A6S7-01 | Tumor | 0.914335665 | 0.650980198 |
| TCGA-DU-A6S8-01 | Tumor | 0.6139758 | 0.588189057 |
| TCGA-DU-A76K-01 | Tumor | 0.795469261 | 0.844146467 |
| TCGA-DU-A76L-01 | Tumor | 1.58114265 | 0.957076019 |
| TCGA-DU-A76O-01 | Tumor | 0.407400192 | 0.701826283 |
| TCGA-DU-A76R-01 | Tumor | 0.623198482 | 0.717004751 |
| TCGA-DU-A7T6-01 | Tumor | 1.081517238 | 0.608209101 |
| TCGA-DU-A7T8-01 | Tumor | 1.008137187 | 0.670456539 |
| TCGA-DU-A7TA-01 | Tumor | 0.793346024 | 0.723571206 |
| TCGA-DU-A7TB-01 | Tumor | 0.766586876 | 0.61369841 |
| TCGA-DU-A7TC-01 | Tumor | 0.775700243 | 0.585724376 |
| TCGA-DU-A7TD-01 | Tumor | 1.152904053 | 0.732631439 |
| TCGA-DU-A7TG-01 | Tumor | 1.077333662 | 0.556880574 |
| TCGA-DU-A7TI-01 | Tumor | 0.708804204 | 0.668011235 |
| TCGA-DU-A7TJ-01 | Tumor | 1.231700093 | 0.904536647 |
| TCGA-E1-5302-01 | Tumor | 0.791009404 | 0.854813614 |
| TCGA-E1-5303-01 | Tumor | 0.914774535 | 0.745560051 |
| TCGA-E1-5304-01 | Tumor | 0.951090271 | 0.768046899 |
| TCGA-E1-5305-01 | Tumor | 0.860728557 | 0.853295113 |
| TCGA-E1-5307-01 | Tumor | 0.809169828 | 0.748869815 |
| TCGA-E1-5311-01 | Tumor | 0.748884729 | 0.989279294 |
| TCGA-E1-5318-01 | Tumor | 0.469510589 | 0.656005557 |
| TCGA-E1-5319-01 | Tumor | 0.543730427 | 0.969275838 |
| TCGA-E1-5322-01 | Tumor | 1.113504896 | 0.861467943 |
| TCGA-E1-A7YD-01 | Tumor | 0.862219026 | 0.878710025 |
| TCGA-E1-A7YE-01 | Tumor | 0.902187164 | 0.916846493 |
| TCGA-E1-A7YH-01 | Tumor | 0.747619312 | 0.737585164 |
| TCGA-E1-A7YI-01 | Tumor | 1.027641115 | 0.916615693 |
| TCGA-E1-A7YJ-01 | Tumor | 1.064817606 | 0.798313 |
| TCGA-E1-A7YK-01 | Tumor | 0.699726076 | 0.566866465 |
| TCGA-E1-A7YL-01 | Tumor | 1.492548606 | 0.981374317 |
| TCGA-E1-A7YM-01 | Tumor | 0.86398198 | 0.766373808 |
| TCGA-E1-A7YN-01 | Tumor | 1.023101802 | 0.720183191 |
| TCGA-E1-A7YO-01 | Tumor | 0.705701271 | 0.861786413 |
| TCGA-E1-A7YQ-01 | Tumor | 1.403629694 | 0.906796722 |
| TCGA-E1-A7YS-01 | Tumor | 0.391307341 | 0.755439131 |
| TCGA-E1-A7YU-01 | Tumor | 0.865391755 | 0.772851953 |
| TCGA-E1-A7YV-01 | Tumor | 0.782945059 | 0.917682955 |
| TCGA-E1-A7YW-01 | Tumor | 1.027693282 | 0.701573128 |
| TCGA-E1-A7YY-01 | Tumor | 1.110680873 | 0.545544405 |
| TCGA-E1-A7Z2-01 | Tumor | 0.728963872 | 0.685846139 |
| TCGA-E1-A7Z3-01 | Tumor | 0.95131231 | 0.804017872 |
| TCGA-E1-A7Z4-01 | Tumor | 0.593582073 | 0.66990542 |
| TCGA-E1-A7Z6-01 | Tumor | 0.781433562 | 0.781015463 |
| TCGA-EZ-7264-01 | Tumor | 0.48712022 | 0.663373477 |
| TCGA-F6-A8O3-01 | Tumor | 0.460781466 | 0.614060254 |
| TCGA-F6-A8O4-01 | Tumor | 0.779986259 | 0.67932973 |
| TCGA-FG-5962-01 | Tumor | 0.710498449 | 0.586287132 |
| TCGA-FG-5963-01 | Tumor | 0.882357818 | 0.656440671 |
| TCGA-FG-5963-02 | Tumor | 1.192544398 | 0.741913939 |
| TCGA-FG-5964-01 | Tumor | 0.622485171 | 0.544103726 |
| TCGA-FG-5965-01 | Tumor | 0.732374234 | 0.739817451 |
| TCGA-FG-5965-02 | Tumor | 0.988357957 | 0.697242767 |
| TCGA-FG-6688-01 | Tumor | 0.89816451 | 0.864212028 |
| TCGA-FG-6689-01 | Tumor | 0.738828253 | 0.644344987 |
| TCGA-FG-6690-01 | Tumor | 0.591339322 | 0.688321067 |
| TCGA-FG-6691-01 | Tumor | 0.942252034 | 0.699125527 |
| TCGA-FG-6692-01 | Tumor | 1.189334943 | 0.787829073 |
| TCGA-FG-7634-01 | Tumor | 1.115322555 | 0.744853872 |
| TCGA-FG-7636-01 | Tumor | 0.79217176 | 0.678309249 |
| TCGA-FG-7637-01 | Tumor | 0.674055202 | 0.728966751 |
| TCGA-FG-7638-01 | Tumor | 0.606582167 | 0.655951912 |
| TCGA-FG-7641-01 | Tumor | 0.78269324 | 0.626976434 |
| TCGA-FG-7643-01 | Tumor | 0.997602207 | 0.670557102 |
| TCGA-FG-8181-01 | Tumor | 1.153090913 | 0.545058967 |
| TCGA-FG-8182-01 | Tumor | 0.657190858 | 0.772857825 |
| TCGA-FG-8185-01 | Tumor | 0.781481761 | 0.73921416 |
| TCGA-FG-8186-01 | Tumor | 0.558230977 | 0.606217554 |
| TCGA-FG-8187-01 | Tumor | 0.534058881 | 0.599298815 |
| TCGA-FG-8188-01 | Tumor | 0.655380867 | 0.742567064 |
| TCGA-FG-8189-01 | Tumor | 1.220458144 | 0.531488839 |
| TCGA-FG-8191-01 | Tumor | 0.870018624 | 0.686760351 |
| TCGA-FG-A4MT-01 | Tumor | 0.666877352 | 0.711929248 |
| TCGA-FG-A4MT-02 | Tumor | 0.888159326 | 0.713349688 |
| TCGA-FG-A4MU-01 | Tumor | 1.681508973 | 1.005556542 |
| TCGA-FG-A4MW-01 | Tumor | 0.947171507 | 0.80474006 |
| TCGA-FG-A4MX-01 | Tumor | 0.420324639 | 0.552977099 |
| TCGA-FG-A4MY-01 | Tumor | 0.398859886 | 0.671428578 |
| TCGA-FG-A60J-01 | Tumor | 0.663065299 | 0.673719897 |
| TCGA-FG-A60K-01 | Tumor | 0.597524924 | 0.607893145 |
| TCGA-FG-A60L-01 | Tumor | 0.682612264 | 0.567990382 |
| TCGA-FG-A6IZ-01 | Tumor | 0.383664694 | 0.561935597 |
| TCGA-FG-A6J1-01 | Tumor | 0.489931053 | 0.53502763 |
| TCGA-FG-A6J3-01 | Tumor | 0.82620866 | 0.630972134 |
| TCGA-FG-A70Y-01 | Tumor | 0.682115925 | 0.732257026 |
| TCGA-FG-A70Z-01 | Tumor | 1.009944431 | 0.785416093 |
| TCGA-FG-A710-01 | Tumor | 0.634925028 | 0.537248691 |
| TCGA-FG-A711-01 | Tumor | 0.835674096 | 0.685017052 |
| TCGA-FG-A713-01 | Tumor | 1.02293003 | 0.585543313 |
| TCGA-FG-A87N-01 | Tumor | 1.056671215 | 0.716035534 |
| TCGA-FG-A87Q-01 | Tumor | 0.995324326 | 1.000570561 |
| TCGA-FN-7833-01 | Tumor | 1.041719534 | 0.765277707 |
| TCGA-HT-7467-01 | Tumor | 0.748008227 | 0.643544809 |
| TCGA-HT-7468-01 | Tumor | 0.481776613 | 0.676409826 |
| TCGA-HT-7469-01 | Tumor | 0.837889714 | 0.995579976 |
| TCGA-HT-7470-01 | Tumor | 0.877715812 | 0.708348234 |
| TCGA-HT-7471-01 | Tumor | 0.75977247 | 0.667062567 |
| TCGA-HT-7472-01 | Tumor | 0.956118422 | 0.85129235 |
| TCGA-HT-7473-01 | Tumor | 0.939659819 | 0.844201544 |
| TCGA-HT-7474-01 | Tumor | 0.93233171 | 0.717453781 |
| TCGA-HT-7475-01 | Tumor | 0.935201252 | 0.703042322 |
| TCGA-HT-7476-01 | Tumor | 1.053449088 | 0.670949663 |
| TCGA-HT-7477-01 | Tumor | 0.848624423 | 0.818784188 |
| TCGA-HT-7478-01 | Tumor | 0.942902589 | 0.641217389 |
| TCGA-HT-7479-01 | Tumor | 0.908020576 | 0.675023998 |
| TCGA-HT-7480-01 | Tumor | 0.637482303 | 0.669667235 |
| TCGA-HT-7481-01 | Tumor | 0.631324277 | 0.902908824 |
| TCGA-HT-7482-01 | Tumor | 0.960256245 | 0.737610952 |
| TCGA-HT-7483-01 | Tumor | 0.857135769 | 0.850887843 |
| TCGA-HT-7485-01 | Tumor | 0.814263038 | 0.771671345 |
| TCGA-HT-7601-01 | Tumor | 0.961936005 | 0.777898039 |
| TCGA-HT-7602-01 | Tumor | 0.784913116 | 0.657481665 |
| TCGA-HT-7603-01 | Tumor | 0.965186219 | 0.70628864 |
| TCGA-HT-7604-01 | Tumor | 0.968415793 | 0.733878651 |
| TCGA-HT-7605-01 | Tumor | 0.85928726 | 0.588833321 |
| TCGA-HT-7606-01 | Tumor | 1.287801662 | 0.758536508 |
| TCGA-HT-7607-01 | Tumor | 0.983721594 | 0.574261515 |
| TCGA-HT-7608-01 | Tumor | 0.721931634 | 0.652543902 |
| TCGA-HT-7609-01 | Tumor | 0.901139074 | 0.792965897 |
| TCGA-HT-7610-01 | Tumor | 0.959670097 | 0.617311693 |
| TCGA-HT-7611-01 | Tumor | 0.830697974 | 0.879776948 |
| TCGA-HT-7616-01 | Tumor | 0.614668016 | 0.775906256 |
| TCGA-HT-7620-01 | Tumor | 0.599403039 | 0.69881085 |
| TCGA-HT-7676-01 | Tumor | 0.801996678 | 0.756425046 |
| TCGA-HT-7677-01 | Tumor | 0.395042608 | 0.564277412 |
| TCGA-HT-7680-01 | Tumor | 0.959706286 | 0.698426708 |
| TCGA-HT-7681-01 | Tumor | 0.864413098 | 0.636568047 |
| TCGA-HT-7684-01 | Tumor | 1.13750465 | 0.575008531 |
| TCGA-HT-7686-01 | Tumor | 0.833270338 | 0.709076498 |
| TCGA-HT-7687-01 | Tumor | 0.627340251 | 0.656267887 |
| TCGA-HT-7688-01 | Tumor | 1.010816936 | 0.726148632 |
| TCGA-HT-7689-01 | Tumor | 0.999412821 | 0.718365933 |
| TCGA-HT-7690-01 | Tumor | 0.966228344 | 0.760042241 |
| TCGA-HT-7691-01 | Tumor | 0.73424486 | 0.546089421 |
| TCGA-HT-7692-01 | Tumor | 0.248563967 | 0.6457885 |
| TCGA-HT-7693-01 | Tumor | 0.732980403 | 0.691298943 |
| TCGA-HT-7694-01 | Tumor | 0.927728692 | 0.786661708 |
| TCGA-HT-7695-01 | Tumor | 0.94947892 | 0.658488779 |
| TCGA-HT-7854-01 | Tumor | 1.028671428 | 0.602231281 |
| TCGA-HT-7855-01 | Tumor | 1.057446416 | 0.861638513 |
| TCGA-HT-7856-01 | Tumor | 0.829788503 | 0.695811606 |
| TCGA-HT-7857-01 | Tumor | 1.14002263 | 0.547371189 |
| TCGA-HT-7858-01 | Tumor | 1.01508178 | 0.711235691 |
| TCGA-HT-7860-01 | Tumor | 1.006625028 | 0.936660363 |
| TCGA-HT-7873-01 | Tumor | 0.819239523 | 0.947802544 |
| TCGA-HT-7874-01 | Tumor | 0.927068937 | 0.743915283 |
| TCGA-HT-7875-01 | Tumor | 0.64552767 | 0.553799208 |
| TCGA-HT-7877-01 | Tumor | 0.768327158 | 0.625299025 |
| TCGA-HT-7879-01 | Tumor | 0.449503086 | 0.871330723 |
| TCGA-HT-7880-01 | Tumor | 0.935780315 | 0.592614011 |
| TCGA-HT-7881-01 | Tumor | 0.93775823 | 0.616813437 |
| TCGA-HT-7882-01 | Tumor | 1.714304814 | 0.809566614 |
| TCGA-HT-7884-01 | Tumor | 0.625432709 | 0.810268269 |
| TCGA-HT-7902-01 | Tumor | 0.692938758 | 0.797644306 |
| TCGA-HT-8010-01 | Tumor | 0.773868396 | 0.588711578 |
| TCGA-HT-8011-01 | Tumor | 1.057722894 | 0.84893066 |
| TCGA-HT-8012-01 | Tumor | 0.465166034 | 0.899336912 |
| TCGA-HT-8015-01 | Tumor | 1.106957548 | 0.588122456 |
| TCGA-HT-8018-01 | Tumor | 1.095307072 | 0.659220353 |
| TCGA-HT-8019-01 | Tumor | 0.980236208 | 0.649453053 |
| TCGA-HT-8104-01 | Tumor | 1.172938613 | 0.896750497 |
| TCGA-HT-8105-01 | Tumor | 0.757108173 | 0.718621437 |
| TCGA-HT-8106-01 | Tumor | 1.131016113 | 0.800497708 |
| TCGA-HT-8107-01 | Tumor | 1.026963598 | 0.559372694 |
| TCGA-HT-8108-01 | Tumor | 0.538163199 | 0.775380465 |
| TCGA-HT-8109-01 | Tumor | 0.868497058 | 0.631058917 |
| TCGA-HT-8110-01 | Tumor | 1.013861052 | 0.762069525 |
| TCGA-HT-8111-01 | Tumor | 0.891175324 | 0.709330274 |
| TCGA-HT-8113-01 | Tumor | 1.07941741 | 0.637233125 |
| TCGA-HT-8114-01 | Tumor | 0.882339448 | 0.906872613 |
| TCGA-HT-8558-01 | Tumor | 1.109665988 | 0.619153731 |
| TCGA-HT-8563-01 | Tumor | 0.865871537 | 1.160090824 |
| TCGA-HT-8564-01 | Tumor | 1.013255436 | 0.657630775 |
| TCGA-HT-A4DS-01 | Tumor | 0.562080728 | 0.580134941 |
| TCGA-HT-A4DV-01 | Tumor | 0.675547737 | 0.640044649 |
| TCGA-HT-A5R5-01 | Tumor | 1.043703581 | 0.772655482 |
| TCGA-HT-A5R7-01 | Tumor | 0.952982066 | 0.61569097 |
| TCGA-HT-A5R9-01 | Tumor | 0.529260547 | 0.667101293 |
| TCGA-HT-A5RA-01 | Tumor | 1.471587074 | 0.978047883 |
| TCGA-HT-A5RB-01 | Tumor | 1.00082639 | 0.759155914 |
| TCGA-HT-A5RC-01 | Tumor | 1.158247525 | 0.843660621 |
| TCGA-HT-A614-01 | Tumor | 0.647890894 | 0.646662632 |
| TCGA-HT-A615-01 | Tumor | 0.573063022 | 0.67007298 |
| TCGA-HT-A616-01 | Tumor | 0.875213271 | 0.627058782 |
| TCGA-HT-A617-01 | Tumor | 0.804637204 | 0.791230951 |
| TCGA-HT-A618-01 | Tumor | 0.912892475 | 0.630253604 |
| TCGA-HT-A619-01 | Tumor | 0.396086754 | 0.689587517 |
| TCGA-HT-A61A-01 | Tumor | 1.088787471 | 0.675805662 |
| TCGA-HT-A61B-01 | Tumor | 0.855612533 | 0.72430145 |
| TCGA-HT-A61C-01 | Tumor | 1.344229241 | 0.628662662 |
| TCGA-HT-A74H-01 | Tumor | 0.692557209 | 0.78426173 |
| TCGA-HT-A74J-01 | Tumor | 0.72600049 | 0.706802091 |
| TCGA-HT-A74K-01 | Tumor | 0.655395578 | 0.480848544 |
| TCGA-HT-A74L-01 | Tumor | 0.492807088 | 0.545374083 |
| TCGA-HT-A74O-01 | Tumor | 0.473938846 | 0.647403953 |
| TCGA-HW-7486-01 | Tumor | 0.557216161 | 0.659680442 |
| TCGA-HW-7487-01 | Tumor | 0.846519583 | 0.84431875 |
| TCGA-HW-7489-01 | Tumor | 0.896816784 | 0.671776823 |
| TCGA-HW-7490-01 | Tumor | 1.027364326 | 0.893600666 |
| TCGA-HW-7491-01 | Tumor | 0.361479831 | 0.697904997 |
| TCGA-HW-7493-01 | Tumor | 1.078846629 | 0.701490495 |
| TCGA-HW-7495-01 | Tumor | 0.816938664 | 0.565536323 |
| TCGA-HW-8319-01 | Tumor | 0.834115294 | 0.717694717 |
| TCGA-HW-8320-01 | Tumor | 1.041562764 | 0.714756879 |
| TCGA-HW-8321-01 | Tumor | 0.513378371 | 0.885423853 |
| TCGA-HW-8322-01 | Tumor | 0.460528197 | 0.595854142 |
| TCGA-HW-A5KJ-01 | Tumor | 0.42216998 | 0.536561776 |
| TCGA-HW-A5KK-01 | Tumor | 1.00173461 | 0.679660192 |
| TCGA-HW-A5KL-01 | Tumor | 0.334471136 | 0.664369141 |
| TCGA-HW-A5KM-01 | Tumor | 1.041098597 | 0.736490588 |
| TCGA-IK-7675-01 | Tumor | 0.747784983 | 0.773735443 |
| TCGA-IK-8125-01 | Tumor | 0.600390414 | 0.593064117 |
| TCGA-KT-A74X-01 | Tumor | 0.376600426 | 0.680548662 |
| TCGA-KT-A7W1-01 | Tumor | 1.315172648 | 0.893718144 |
| TCGA-P5-A5ET-01 | Tumor | 0.629780386 | 0.630089816 |
| TCGA-P5-A5EU-01 | Tumor | 1.353292978 | 0.872504143 |
| TCGA-P5-A5EV-01 | Tumor | 0.726934059 | 0.741028696 |
| TCGA-P5-A5EW-01 | Tumor | 0.846276714 | 0.808628537 |
| TCGA-P5-A5EX-01 | Tumor | 0.960990767 | 0.616791672 |
| TCGA-P5-A5EY-01 | Tumor | 0.657331847 | 0.610081182 |
| TCGA-P5-A5EZ-01 | Tumor | 0.83964298 | 0.745350385 |
| TCGA-P5-A5F0-01 | Tumor | 0.820956206 | 0.496262319 |
| TCGA-P5-A5F1-01 | Tumor | 0.773730199 | 0.69457509 |
| TCGA-P5-A5F2-01 | Tumor | 0.827194342 | 0.993887774 |
| TCGA-P5-A5F4-01 | Tumor | 0.946882638 | 0.743471209 |
| TCGA-P5-A5F6-01 | Tumor | 1.354418925 | 0.8556403 |
| TCGA-P5-A72U-01 | Tumor | 0.809204127 | 0.682784782 |
| TCGA-P5-A72W-01 | Tumor | 0.798029385 | 0.678197353 |
| TCGA-P5-A72X-01 | Tumor | 0.784589092 | 0.713467989 |
| TCGA-P5-A72Z-01 | Tumor | 0.296579612 | 0.637133745 |
| TCGA-P5-A730-01 | Tumor | 0.405195042 | 0.693884642 |
| TCGA-P5-A731-01 | Tumor | 1.003215832 | 0.747487552 |
| TCGA-P5-A733-01 | Tumor | 0.396066364 | 0.659299867 |
| TCGA-P5-A735-01 | Tumor | 0.57080256 | 0.654729133 |
| TCGA-P5-A736-01 | Tumor | 0.566575974 | 0.773406406 |
| TCGA-P5-A737-01 | Tumor | 0.503169268 | 0.542958065 |
| TCGA-P5-A77W-01 | Tumor | 0.384634193 | 0.612507941 |
| TCGA-P5-A77X-01 | Tumor | 0.77479292 | 0.74469923 |
| TCGA-P5-A780-01 | Tumor | 1.037839924 | 0.822431989 |
| TCGA-P5-A781-01 | Tumor | 0.499569327 | 0.817670682 |
| TCGA-QH-A65R-01 | Tumor | 0.588619408 | 0.826017544 |
| TCGA-QH-A65S-01 | Tumor | 0.511131376 | 0.613361875 |
| TCGA-QH-A65V-01 | Tumor | 0.658769673 | 0.616495035 |
| TCGA-QH-A65X-01 | Tumor | 0.573984159 | 0.671805436 |
| TCGA-QH-A65Z-01 | Tumor | 0.450540595 | 0.584409204 |
| TCGA-QH-A6CS-01 | Tumor | 0.793271683 | 0.707737874 |
| TCGA-QH-A6CU-01 | Tumor | 0.42728872 | 0.5998959 |
| TCGA-QH-A6CV-01 | Tumor | 0.921669254 | 0.835406671 |
| TCGA-QH-A6CW-01 | Tumor | 0.770684041 | 0.649589614 |
| TCGA-QH-A6CX-01 | Tumor | 0.780247445 | 0.811752816 |
| TCGA-QH-A6CY-01 | Tumor | 0.959581889 | 0.626633816 |
| TCGA-QH-A6CZ-01 | Tumor | 0.693112798 | 0.590477449 |
| TCGA-QH-A6X3-01 | Tumor | 0.446897002 | 0.804859007 |
| TCGA-QH-A6X4-01 | Tumor | 0.400042958 | 0.722022016 |
| TCGA-QH-A6X5-01 | Tumor | 0.443125879 | 0.592824344 |
| TCGA-QH-A6X8-01 | Tumor | 0.434257304 | 0.67435033 |
| TCGA-QH-A6X9-01 | Tumor | 0.5160156 | 0.764382685 |
| TCGA-QH-A6XA-01 | Tumor | 0.560920206 | 0.663644177 |
| TCGA-QH-A6XC-01 | Tumor | 0.958882119 | 0.7146309 |
| TCGA-QH-A86X-01 | Tumor | 0.534381443 | 0.537277312 |
| TCGA-QH-A870-01 | Tumor | 0.404234766 | 0.746235717 |
| TCGA-R8-A6MK-01 | Tumor | 0.416718959 | 0.583187601 |
| TCGA-R8-A6ML-01 | Tumor | 0.353961198 | 0.614830991 |
| TCGA-R8-A6MO-01 | Tumor | 0.525936039 | 0.625162591 |
| TCGA-R8-A6YH-01 | Tumor | 0.942009336 | 0.804195298 |
| TCGA-R8-A73M-01 | Tumor | 0.377169991 | 0.501025109 |
| TCGA-RY-A83X-01 | Tumor | 0.345100354 | 0.646842484 |
| TCGA-RY-A83Y-01 | Tumor | 0.384874041 | 0.510414304 |
| TCGA-RY-A83Z-01 | Tumor | 0.691874815 | 0.609757161 |
| TCGA-RY-A840-01 | Tumor | 0.315547896 | 0.554883848 |
| TCGA-RY-A843-01 | Tumor | 0.634642605 | 0.598173066 |
| TCGA-RY-A845-01 | Tumor | 0.751714174 | 0.728322043 |
| TCGA-RY-A847-01 | Tumor | 0.648556395 | 0.623841631 |
| TCGA-S9-A6TS-01 | Tumor | 0.892544843 | 0.742907404 |
| TCGA-S9-A6TU-01 | Tumor | 0.61977766 | 0.725259873 |
| TCGA-S9-A6TV-01 | Tumor | 1.246873117 | 0.93182685 |
| TCGA-S9-A6TW-01 | Tumor | 0.490579462 | 0.737173219 |
| TCGA-S9-A6TX-01 | Tumor | 0.541197536 | 0.580000637 |
| TCGA-S9-A6TY-01 | Tumor | 0.598354036 | 0.610376999 |
| TCGA-S9-A6TZ-01 | Tumor | 0.897168221 | 0.777342848 |
| TCGA-S9-A6U0-01 | Tumor | 1.289889483 | 0.927611513 |
| TCGA-S9-A6U1-01 | Tumor | 0.951842155 | 0.715691591 |
| TCGA-S9-A6U2-01 | Tumor | 0.547417253 | 0.589150865 |
| TCGA-S9-A6U5-01 | Tumor | 0.414072178 | 0.528774819 |
| TCGA-S9-A6U6-01 | Tumor | 0.561823517 | 0.77582726 |
| TCGA-S9-A6U8-01 | Tumor | 0.54215358 | 0.684511776 |
| TCGA-S9-A6U9-01 | Tumor | 0.781251343 | 0.772337748 |
| TCGA-S9-A6UA-01 | Tumor | 1.09415364 | 0.629941919 |
| TCGA-S9-A6UB-01 | Tumor | 0.375930291 | 0.586381685 |
| TCGA-S9-A6WD-01 | Tumor | 0.457960793 | 0.708901173 |
| TCGA-S9-A6WE-01 | Tumor | 0.625020671 | 0.64414011 |
| TCGA-S9-A6WG-01 | Tumor | 1.141964324 | 0.688095466 |
| TCGA-S9-A6WH-01 | Tumor | 0.458683448 | 0.722705187 |
| TCGA-S9-A6WI-01 | Tumor | 1.052767808 | 0.538956089 |
| TCGA-S9-A6WL-01 | Tumor | 0.809113528 | 0.861481095 |
| TCGA-S9-A6WM-01 | Tumor | 0.911523997 | 0.850084198 |
| TCGA-S9-A6WN-01 | Tumor | 0.627015785 | 0.807570266 |
| TCGA-S9-A6WO-01 | Tumor | 0.90613629 | 0.664177347 |
| TCGA-S9-A6WP-01 | Tumor | 0.466319218 | 0.632597843 |
| TCGA-S9-A7IQ-01 | Tumor | 1.072199005 | 0.483991704 |
| TCGA-S9-A7IS-01 | Tumor | 1.325208838 | 0.757429207 |
| TCGA-S9-A7IX-01 | Tumor | 1.095449928 | 0.749619557 |
| TCGA-S9-A7IY-01 | Tumor | 0.642329276 | 0.675293208 |
| TCGA-S9-A7IZ-01 | Tumor | 0.663916073 | 0.685547817 |
| TCGA-S9-A7J0-01 | Tumor | 0.657906589 | 0.809333114 |
| TCGA-S9-A7J1-01 | Tumor | 0.389224204 | 0.555528659 |
| TCGA-S9-A7J2-01 | Tumor | 0.488364525 | 0.620676672 |
| TCGA-S9-A7J3-01 | Tumor | 0.54352387 | 0.712757646 |
| TCGA-S9-A7QW-01 | Tumor | 0.793865034 | 0.604927756 |
| TCGA-S9-A7QX-01 | Tumor | 0.737548791 | 0.775790613 |
| TCGA-S9-A7QY-01 | Tumor | 0.473688102 | 0.537772397 |
| TCGA-S9-A7QZ-01 | Tumor | 0.548340255 | 0.562174034 |
| TCGA-S9-A7R1-01 | Tumor | 0.539768401 | 0.639316194 |
| TCGA-S9-A7R2-01 | Tumor | 0.792012435 | 0.714799975 |
| TCGA-S9-A7R3-01 | Tumor | 0.433304637 | 0.663170642 |
| TCGA-S9-A7R4-01 | Tumor | 0.799971701 | 0.758616089 |
| TCGA-S9-A7R7-01 | Tumor | 0.698703928 | 0.779362421 |
| TCGA-S9-A7R8-01 | Tumor | 0.803352251 | 0.843347658 |
| TCGA-S9-A89V-01 | Tumor | 0.952785211 | 0.553181094 |
| TCGA-S9-A89Z-01 | Tumor | 0.533601302 | 0.864637238 |
| TCGA-TM-A7C3-01 | Tumor | 0.870613984 | 0.872191419 |
| TCGA-TM-A7C4-01 | Tumor | 0.794070015 | 0.673505902 |
| TCGA-TM-A7C5-01 | Tumor | 0.487244354 | 0.655396564 |
| TCGA-TM-A7CA-01 | Tumor | 0.648012202 | 0.690715182 |
| TCGA-TM-A7CF-01 | Tumor | 0.86293977 | 0.646546895 |
| TCGA-TM-A7CF-02 | Tumor | 0.735623822 | 0.649703574 |
| TCGA-TM-A84B-01 | Tumor | 1.009462894 | 0.840287234 |
| TCGA-TM-A84C-01 | Tumor | 1.274096142 | 0.681396617 |
| TCGA-TM-A84F-01 | Tumor | 0.793476131 | 0.675079796 |
| TCGA-TM-A84G-01 | Tumor | 0.695189619 | 0.64545403 |
| TCGA-TM-A84H-01 | Tumor | 0.674675009 | 0.571552659 |
| TCGA-TM-A84I-01 | Tumor | 0.810358935 | 0.838922051 |
| TCGA-TM-A84J-01 | Tumor | 1.409318173 | 0.846096978 |
| TCGA-TM-A84L-01 | Tumor | 0.408958359 | 0.760494376 |
| TCGA-TM-A84M-01 | Tumor | 0.345650675 | 0.585444396 |
| TCGA-TM-A84O-01 | Tumor | 0.442898353 | 0.851196164 |
| TCGA-TM-A84Q-01 | Tumor | 0.911556167 | 0.757615756 |
| TCGA-TM-A84R-01 | Tumor | 1.006370777 | 0.547751764 |
| TCGA-TM-A84S-01 | Tumor | 0.431357253 | 0.528807615 |
| TCGA-TM-A84T-01 | Tumor | 0.886050587 | 0.78665817 |
| TCGA-TQ-A7RF-01 | Tumor | 0.920109702 | 0.766261624 |
| TCGA-TQ-A7RG-01 | Tumor | 0.597556534 | 0.604156602 |
| TCGA-TQ-A7RH-01 | Tumor | 0.52120694 | 0.716564167 |
| TCGA-TQ-A7RI-01 | Tumor | 0.653444913 | 0.572756324 |
| TCGA-TQ-A7RJ-01 | Tumor | 0.934979654 | 0.898568061 |
| TCGA-TQ-A7RK-01 | Tumor | 0.695772048 | 0.67394957 |
| TCGA-TQ-A7RK-02 | Tumor | 0.962706762 | 0.718544116 |
| TCGA-TQ-A7RM-01 | Tumor | 1.257993281 | 0.844307955 |
| TCGA-TQ-A7RN-01 | Tumor | 0.529098259 | 0.639258506 |
| TCGA-TQ-A7RO-01 | Tumor | 0.730766993 | 0.763297731 |
| TCGA-TQ-A7RP-01 | Tumor | 0.897724176 | 0.768999082 |
| TCGA-TQ-A7RQ-01 | Tumor | 0.510690955 | 0.741629459 |
| TCGA-TQ-A7RR-01 | Tumor | 0.710442325 | 0.686649343 |
| TCGA-TQ-A7RS-01 | Tumor | 0.329292102 | 0.60378854 |
| TCGA-TQ-A7RU-01 | Tumor | 0.332796464 | 0.629006938 |
| TCGA-TQ-A7RV-01 | Tumor | 0.706828708 | 0.853349188 |
| TCGA-TQ-A7RV-02 | Tumor | 0.823412735 | 0.744952951 |
| TCGA-TQ-A7RW-01 | Tumor | 0.813951928 | 0.969677712 |
| TCGA-TQ-A8XE-01 | Tumor | 0.403642039 | 0.78804984 |
| TCGA-TQ-A8XE-02 | Tumor | 0.492070932 | 0.805316894 |
| TCGA-VM-A8C8-01 | Tumor | 0.818242154 | 0.636101885 |
| TCGA-VM-A8C9-01 | Tumor | 0.837589253 | 0.451693631 |
| TCGA-VM-A8CA-01 | Tumor | 0.774452704 | 0.768506129 |
| TCGA-VM-A8CB-01 | Tumor | 0.403694346 | 0.827626647 |
| TCGA-VM-A8CD-01 | Tumor | 1.257553681 | 0.891031575 |
| TCGA-VM-A8CE-01 | Tumor | 0.603759478 | 0.552138681 |
| TCGA-VM-A8CF-01 | Tumor | 0.410711458 | 0.567000206 |
| TCGA-VM-A8CH-01 | Tumor | 0.918281062 | 0.693750232 |
| TCGA-VV-A829-01 | Tumor | 0.473849431 | 0.552503429 |
| TCGA-VV-A86M-01 | Tumor | 1.05045095 | 0.750815917 |
| TCGA-VW-A7QS-01 | Tumor | 0.416303529 | 0.803723163 |
| TCGA-VW-A8FI-01 | Tumor | 0.799243157 | 0.734000826 |
| TCGA-W9-A837-01 | Tumor | 0.686363683 | 0.545317137 |
| TCGA-WH-A86K-01 | Tumor | 0.328972083 | 0.62686809 |
| TCGA-WY-A858-01 | Tumor | 0.670232172 | 0.609353917 |
| TCGA-WY-A859-01 | Tumor | 0.932678702 | 0.619023484 |
| TCGA-WY-A85A-01 | Tumor | 0.812390208 | 0.632285598 |
| TCGA-WY-A85B-01 | Tumor | 0.75519064 | 0.731431906 |
| TCGA-WY-A85C-01 | Tumor | 0.904732306 | 0.961633209 |
| TCGA-WY-A85D-01 | Tumor | 0.602271603 | 0.66630482 |
| TCGA-WY-A85E-01 | Tumor | 0.682955094 | 0.684405415 |
