# Supplementary figures and images for: Identification and validation of LDHA and SLC16A1 for predicting prognosis and diagnosis in lower-grade glioma
Source: Discov Oncol. 2025 Aug 9;16:1511. doi: 10.1007/s12672-025-03297-2 (PMC12335421; doi:10.1007/s12672-025-03297-2)

Full unedited gel/blot for Fig 9B

1. SW1088 cells

SLC16A1


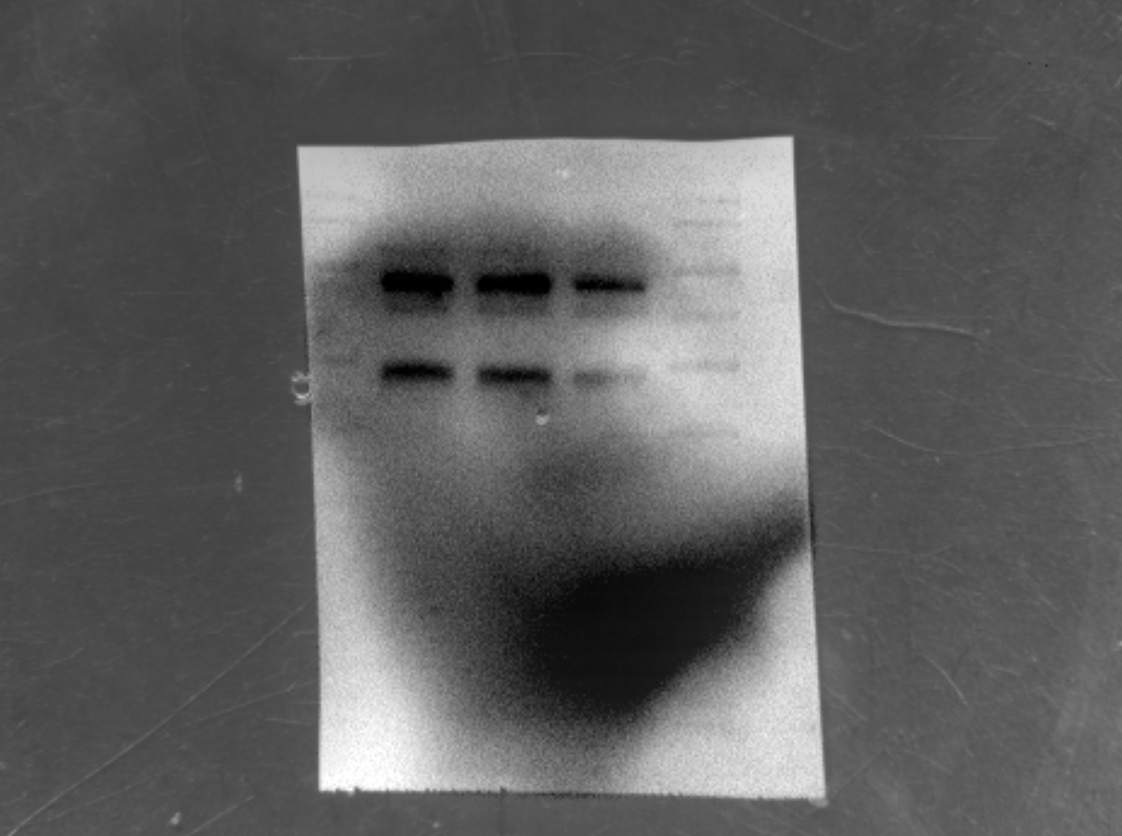


GAPDH


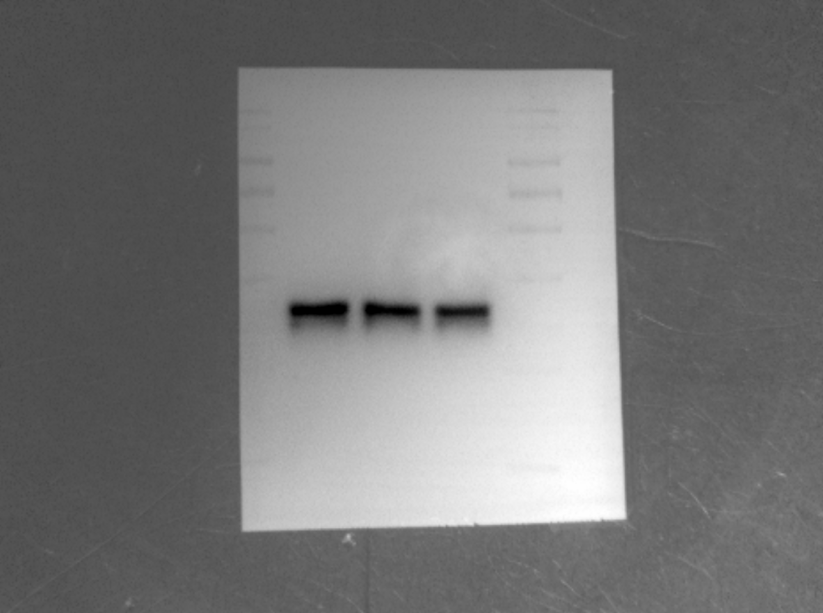


1. HS683 cells

SLC16A1


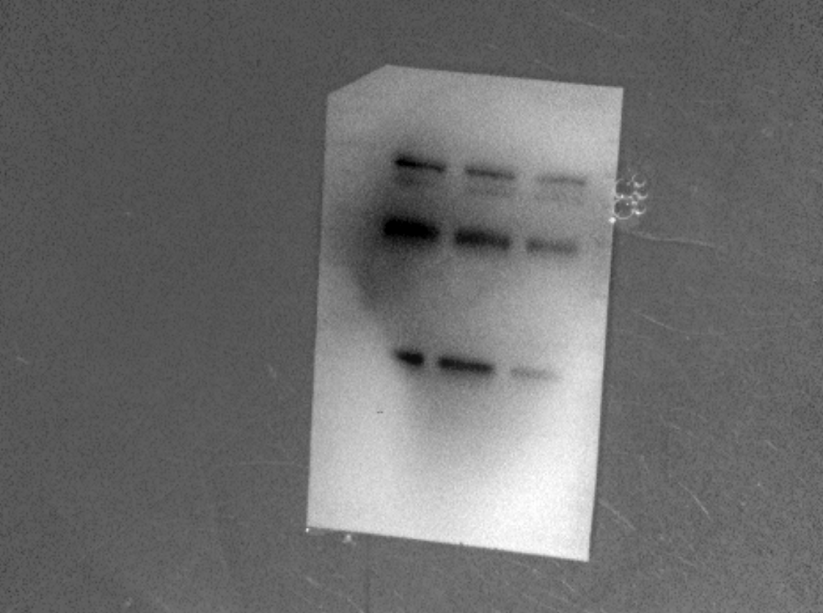


GAPDH


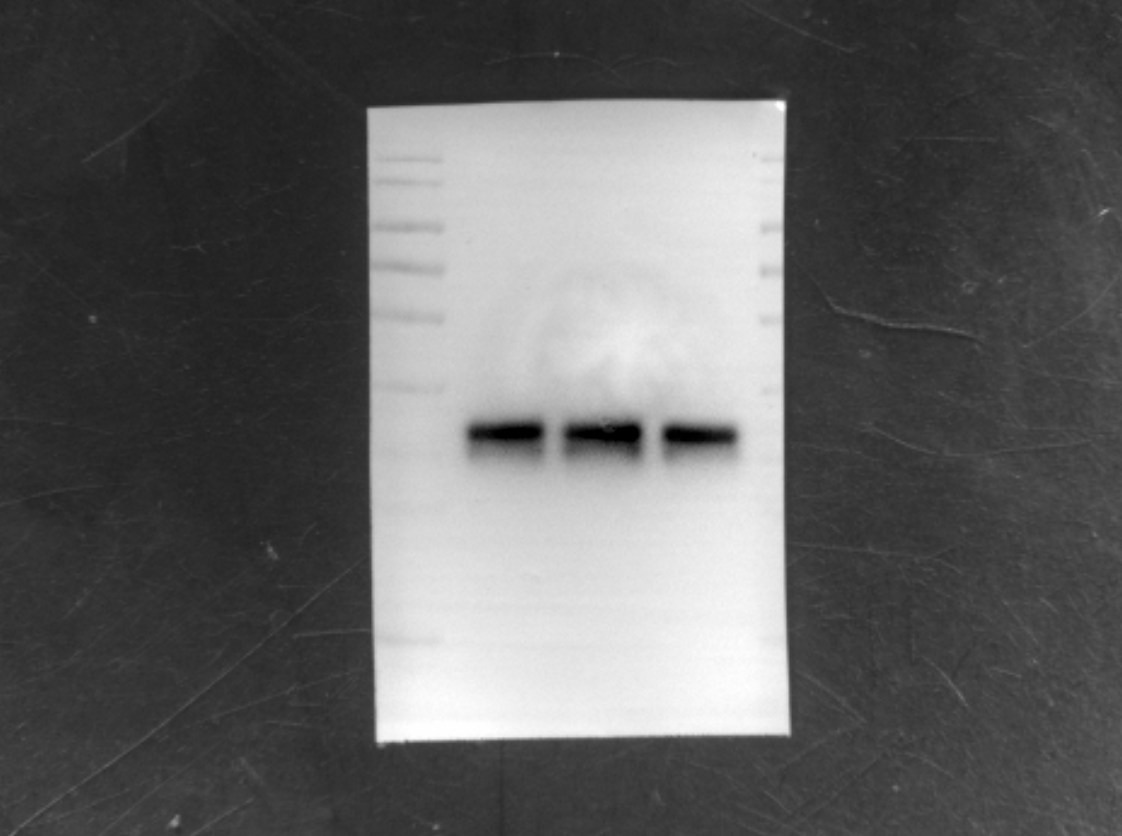

Supplement: Supplementary file 3 — Supplementary Material 3. [file 12672_2025_3297_MOESM3_ESM.docx]
